# Generative Participatory Design Methodology to Develop Electronic Health Interventions: Systematic Literature Review

**DOI:** 10.2196/13780

**Published:** 2020-04-27

**Authors:** Pieter Vandekerckhove, Marleen de Mul, Wichor M Bramer, Antoinette A de Bont

**Affiliations:** 1 Erasmus School of Health Policy and Management Rotterdam Netherlands; 2 Medical Library Erasmus MC Erasmus Medical Center Rotterdam Rotterdam Netherlands

**Keywords:** cocreation, co-design, participatory design, telemedicine, eHealth, medical informatics, method, methodology, review

## Abstract

**Background:**

Generative participatory design (PD) may help in developing electronic health (eHealth) interventions. PD is characterized by the involvement of all stakeholders in creative activities. This is different from the traditional user-centered design, where users are less involved. When looking at PD from a *research through design* perspective, it is important to summarize the reasons for choosing a certain form of generative PD to further develop its methodology. However, the scientific literature is currently unclear about which forms of PD are used to develop eHealth and which arguments are used to substantiate the decision to use a certain form of generative PD.

**Objective:**

This study aimed to explore the reporting and substantiation of generative PD methodologies in empirical eHealth studies published in scientific journals to further develop PD methodology in the field of eHealth.

**Methods:**

A systematic literature review following the Cochrane guidelines was conducted in several databases (EMBASE, MEDLINE Ovid, Web of Science, and CINAHL EBSCOhost). Data were extracted on the recruitment and management of stakeholders, the use of tools, and the use of outcome measures.

**Results:**

Of the 3131 studies initially identified, 69 were selected for qualitative synthesis. The reporting was very variable, depending to a large extent on whether the study stated that reporting on the PD process was a major aim. The different levels of reporting and substantiation of the choices of a recruitment strategy, stakeholder management, and tools and outcome measures are presented. Only a few authors explicitly used arguments directly related to PD guiding principles such as democratic, mutual learning, tacit and latent knowledge, and collective creativity. Even though PD principles were not always explicitly discussed in the method descriptions of the studies, they were implicitly present, mostly in the descriptions of the use of PD tools. The arguments used to substantiate the choices made in stakeholder management, PD tools, and the type of outcome measures adopted point to the involvement of PD principles.

**Conclusions:**

Studies that have used a PD research methodology to develop eHealth primarily substantiate the choice of tools made and much less the use of stakeholders and outcome measures.

## Introduction

### Participatory Design Methodology

Stakeholder participation is considered to play an important role in developing electronic health interventions (eHealth) [1-4]. However, during the development of eHealth, challenges remain in gaining the trust of stakeholders, managing multiple stakeholders, and involving end users [1,5]. In contrast to more traditional forms of user-centered design, where stakeholders are less involved, generative participatory design (PD) focuses on including stakeholders in creative activities [3,4]. Therefore, PD is promising in that it could overcome the challenges seen in the development of eHealth [6-8].

PD is becoming increasingly intertwined with research and is therefore also considered to be a research methodology. Looking at PD from a research perspective, the methodological choices to be made are of particular interest. Methodological elements that play a key role in PD research are the recruitment and management of stakeholders [4], the use of outcome measures [4], and the use of tools [9,10]. The literature indicates that the application of each of these elements varies when PD is employed.

Looking at the literature on participatory methods to develop eHealth, a recent systematic literature review showed that 24 frameworks have been used [11]. However, as many studies do not refer to a framework, more attention is needed on the methodologies employed [11].

### Methodological Elements

Turning to stakeholders, the varying involvement of patients as end users has been widely discussed in the literature [12,13]. Warnings have been given regarding the ability of users to express their needs and about the prejudices of PD practitioners regarding the participants [14], and the involvement of end users remains debated. When it comes to outcome measures, there is a wide variety that can be used to evaluate PD outputs related to the PD process itself and to the eHealth technology output [15,16]. Tools describe the actions that take place between participants [17], and PD scholars have categorized these tools into make, tell, and enact tools [3,10,17]. Make tools are material components such as a prototype to facilitate the embodiment of thoughts in physical artifacts [10]. Tell tools facilitate the telling of stories to capture implicit information about the use of a technology and how people may wish to use it in the future [10]. Enacting refers to the activities where one or more people act out possible futures by physically trying things out in settings that resemble the possible futures [10]. Finally, PD toolkits can involve make, tell, and enact tools and are used to push people to start thinking about their experiences so that using the tools in the PD process can yield better results.

How stakeholders, tools, and outcome measures are employed in the PD process depends on which PD methodology is followed. Furthermore, there is a lack of a strong methodological explanation that could help develop a more rigorous science of PD [2,4]. Using methodological arguments to make each methodological decision applied in studies employing PD more explicit could improve the scientific rigor of PD as a research methodology [18].

### Guiding Principles

The PD literature encompasses various theories that form the foundations for methodologies [2-4,9,10]. Value-laden concepts such as democracy, participation, empowerment, and empathy [4,9] contain values such as inclusion and equality [9] and play a fundamental role in PD. On the basis of the work by Van der Velden and Mörtberg [9] and of Sanders and Stappers [3], four key guiding PD principles can be discerned:

- Democracy: In contrast to traditional design practices, the aim is to involve all stakeholders including nondesigners and future users who will be affected by new technologies. Users can become part of the design team as *experts of their experiences* given appropriate tools to express themselves [13]. The aim is to increase diversity of experience, values, and knowledge. This is believed to foster trust among those involved and to facilitate a learning process and a commitment to taking responsibility for each other and the design result.
- Mutual learning: Participants (both designers and nondesigners) learn from each other, but they also learn from themselves when reflecting on their own work.
- Tacit or latent knowledge: To assess the needs of people beyond the observable or easily detectable, that is, in the form of tacit needs. This deeper knowledge includes explicit and implicit day-to-day technological expertise from the present, future, and past [19]. Sanders has defined tacit needs as being conscious but not expressed and latent needs as subconscious needs that cannot be expressed in words [3,20].
- Collective creativity: PD is considered to be essentially a process of collective creativity [3]. Sanders and Stappers [3] refer to social creativity in which people follow a process referred to as *the path of expression*. Creativity facilitates a design process from which values emerge and become inscribed in the product or service [9]. Everyone is assumed to possess some creative ability, although a design role requires a certain level of creativity [13].

Given the developing nature of the PD methodology, the theoretical and empirical literature does not always incorporate these insights and the four guiding principles. In the theoretical design literature, the relationship between PD principles and the use of stakeholders, tools, and outcome measures is only implicitly suggested [2,4]. For instance, PD principles seem to be implicit in the description of make tools. The democratic principle is implicitly present as make tools include both designers and nondesigners in *making things* [10]. As such, make tools can be used to enhance the democratic involvement of stakeholders. In addition, the collective creativity principle is also implicitly present. Tools, depending on the aim, can be used within a PD project to (1) probe participants, (2) prime participants—to immerse them in a domain, (3) to gain a better understanding of their experiences, or (4) to generate new ideas [17]. Depending on the aim, make tools can be used as part of a probing approach (to inspire ideas), a participatory prototyping approach (stakeholders provide feedback on an existing prototype), or a generative approach (stakeholders give ideas a physical form) [10,19]. It has been suggested that the probing and generative approaches are better suited to early design, or the so-called fuzzy front end, and that prototyping is more useful in later, less fuzzy, design stages [19]. Therefore, the democratic principle and the creativity principles can be used to argue in favor of adopting make tools at different times in the design process.

Little has been reported on the specific arguments used to explain the choice of specific stakeholders, tools, and outcome measures. Although stakeholders can be involved in various ways in the development of gerontology [8], mobile health (mHealth) [7], and serious games [6,21], a discussion on the methodological considerations is missing. Second, various tools are described for developing health information technology [22], gerontology [8], and mHealth [7], but without methodological substantiation.

In addition, given the very limited presence of evaluations in the empirical literature, it is difficult to establish the outcome measures that are used, let alone the principles upon which they are selected. Eyles et al [7] failed to find any mHealth studies that reported outcome measures. Merkel and Kucharski [8] found a few studies that evaluated some eHealth results, for example, by testing a prototype. However, they did not report the results of the evaluations [8]. Merkel and Kucharski [8] also stated that there were no studies that had evaluated the process of PD itself. Exceptionally, DeSmet et al [21] did evaluate the effectiveness of PD in serious games. They expected that the use of PD in the development of serious games was less effective than when users were involved merely as testers in the game (albeit without taking sample size and strength of effect into account) [21].

### Aim

Given these uncertainties, the aim of this study was to explore the substantiation behind the methodological choice to use a certain form of PD in developing eHealth. This paper was intended to be a start in looking at the state of reporting of PD research methodology and, therefore, used a systematic literature review to summarize the current status of reporting in peer-reviewed scientific journals. This research has the potential to guide researchers and practitioners to areas where greater substantiation is needed when using or reporting PD. By considering the current methodological choices, some recommendations are also provided that may also help researchers and practitioners select a method that helps them better achieve their aims.

## Methods

### Systematic Literature Review

A systematic literature review with qualitative synthesis was conducted to summarize existing knowledge on PD methodology in the development of eHealth technology. In the medical field, the Cochrane review process is considered the gold standard. Given that this review is focused on eHealth, this systematic review follows the Cochrane guidelines [23]. To ensure completeness and transparency, a Preferred Reporting Items for Systematic Reviews and Meta-Analyses (PRISMA) reporting statement is included [24].

Given that PD methodology is developing, the focus was on the reported use and justification of the choices made in using PD tools, stakeholder management, stakeholder recruitment, and the outcome measures selected. The first research question focuses on the use of PD: “How is the use of PD, in particular the involvement of stakeholders, the use of tools, and the use of outcome measures described in the empirical literature about eHealth development?” The second research question focuses on the justification for a type of PD: “What reasons, related to the guiding principles of PD, are offered to substantiate the preference for a given use of stakeholders, tools, and outcome measures?”

### Selection of Studies

Search queries were developed by an experienced medical information specialist (WB) and the searches used terms such as participatory design, co-design, cocreation, and collaborative design in the field of telehealth. In addition to these terms, we used a more descriptive approach where we combined human centeredness, patient involvement, etc, with shared decision making or doctor-patient relations in the field of telehealth. The term user involvement was also added to the search. The term participatory research was not used as the terms “co-creation,” “co-design,” and “participatory” were assumed to cover this field.

The search strategies for all the databases that were used can be found in Multimedia Appendix 1. The following databases have been searched from their inception until November 12, 2019 (date last searched): EMBASE (1974-), MEDLINE ALL (Ovid, 1946-), Web of Science Core Collection (Web of Knowledge, 1900-), and CINAHL (EBSCOhost, 1937-). All the references from searches on electronic databases were exported and duplicates removed in Endnote X9 (Thompson Reuters Inc) software. The identified titles and abstracts were then screened for eligibility by two independent researchers.

The following working definition for PD was used: PD refers to the collective creative design process of designers and nondesigners, whereby users are considered partners during the design process. PD activities can generally be described as cocreation workshops or cocreation exercises, or they can be more specifically described by referring to make (ie, collage), tell (ie, cards), and act (ie, acting out) tools. Studies that used other terms were also included if they were described by the authors as co-design or PD-related activities [10,17]. Studies that used other popular terms such as cocreation were only included if, as part of the methodology, PD tools were described.

The selection criteria for inclusion and exclusion are shown in Textboxes 1 and 2. Studies that had as their main objective developing eHealth technology were included. Articles in conference proceedings were also included. Study protocols and conference abstracts were excluded as these included insufficient information about the execution of the PD study and its results. Non-English language publications were excluded.

All types of empirical study designs were included, and no restrictions were placed on the types of participants. For instance, studies involving only patients or only care professionals in PD were included. The presence of PD activities was chosen as the inclusion criterion rather than other features of PD because this area has the most clearly defined consensus in the literature. Other aspects of PD, such as stakeholder recruitment, stakeholder management, and PD outcome measures, were not used as the inclusion or exclusion criteria as these terms can be used in somewhat arbitrary ways.

**Inclusion criteria for screening.**

- Language: English language
- Format: Full text available (including full conference papers)
- Study design: Empirical study describing the direct or indirect observation or experience of using participatory design (PD) to develop electronic health (eHealth) published in a peer-reviewed journal or conference proceedings. The aim of the paper was to report on the use of PD to develop eHealth.
- Product or service developed: eHealth related
- Method of development: PD as a collective creative design process of designers and nondesigners whereby users are considered to be partners in the process and the use of PD activities is described with this mindset (including participatory prototyping)
- Design development phases: All innovation phases included (predesign, early design [discover], and design and make)
- Setting: at least one of the PD tools used must be in a group setting (ie, more than one individual involved)

**Exclusion criteria for screening.**

- Language: Other than English
- Format: Only abstract or full text unavailable
- Study design: Nonempirical studies (ie, reviews, editorials, discussion papers, methodological papers, papers reflecting on eHealth developed with PD), studies not peer reviewed (eg, dissertations)
- Product or service developed: Other than electronic health (eHealth
- Method of development: Nonparticipatory design, participatory design (PD) where users are considered as subjects in the design process (user-centered design), the use of PD is not described (ie, only qualitative research tools such as focus groups or interviews)
- Setting: All PD tools used only by individuals
- Design development phases: Value cocreation excluded (market phase and later marketing phases)

**Figure 1:**
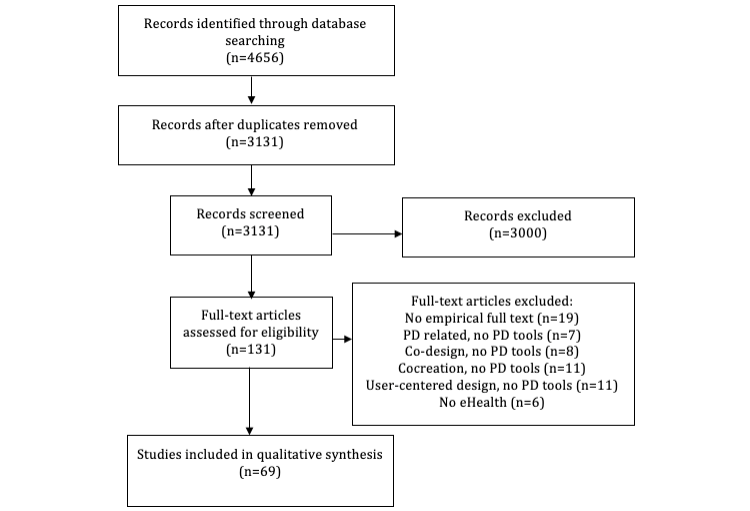
Preferred Reporting Items for Systematic Reviews and Meta-Analyses flow diagram. eHealth: electronic health; PD: participatory design.

The identification and selection of studies is summarized in Figure 1 according to the PRISMA guidelines [24]. Following the removal of duplicates, 3131 articles were identified through the search strategy, of which 3000 articles were then excluded based on the title and contents of the abstract. This left 131 unique full-text studies for review, of which 69 met the inclusion criteria (see Multimedia Appendix 2 for full-text studies excluded). The main reasons for full-text exclusion were (1) not considered to be empirical studies or full-text peer-reviewed documents (eg, conference abstracts, protocols, and a PhD thesis; n=19), (2) mentioned PD-related activities, but no PD tools (n=7), (3) mentioned co-design but no PD tools (n=8), (4) mentioned cocreation but no PD tools (n=11), (5) mentioned user-centered design, but no PD tools (n=11), and (6) did not mention eHealth (n=6).

### Data Extractions and Synthesis

To provide an overview of the general characteristics of the studies, the eHealth domain, the health domain, and the theoretical references used to refer to PD were summarized. In addition, the use of stakeholders, tools, and outcome measures was assessed as follows.

First, regarding the use of stakeholders, different strategies could be used depending on the interpretation of PD principles. Therefore, data were extracted related to the number and type of stakeholders, stakeholder recruitment, and stakeholder management. Second, regarding the use of tools, different tools can be used at different times depending on the PD principles. Therefore, the type of tool and the purpose in using the tool were extracted. Finally, the study was placed in a design phase depending on the stage in which the study started: predesign, early design, or post first prototype (Table 1).

**Table 1:** Description of design phases.

Design phase	Description
Predesign (including fuzzy front end)	Phase of understanding and defining the problem, often these studies would focus on the unmet need of a certain population.
Early design	In this phase, there is already some understanding of the problem and the aim is to develop a first concrete idea, often these studies would aim to develop or enhance a first idea or prototype.
Post first prototype	In this phase, there is already a first idea for a solution, which will be iterated or enhanced.

Third, as the use of outcome measures is influenced by the general methodological aim and the principles that are emphasized, the type of outcome measure that was used to evaluate eHealth and the PD itself was extracted.

### Assessment of Sufficiency of Reporting

Owing to the variety of study designs, a quality assessment was not appropriate. Instead, an assessment of sufficiency of reporting was conducted, as used in a previous systematic review [7]. This was done with an 8-item checklist:

1. Setting: Is it clear where the PD development of the intervention took place?
2. Stakeholders: Is it clear who was involved in the PD, and does one know all that one needs to know about the participants?
3. Facilitators: Is it clear who facilitated the PD process?
4. Procedure: Is it clear what PD methods were used?
5. Materials: Are any physical materials used in the PD process adequately described?
6. Intensity: Is the length of the PD phase and individual sessions clear?
7. Schedule: Is the interval and frequency of the PD sessions clear?
8. Clarity: Is the description of the overall PD process clear?

## Results

### Overall Findings

The general health and eHealth technology characteristics and the theoretical references used in the studies when referring to PD are described below. The year of publication ranged from 2006 to 2019. The 69 studies cover 65 unique eHealth technology products and services. The majority of these were either Web-based tools such as *online self-management tools* [25], *person-centered Web support* [26], or a *Web-based plan for integrated care* [27], or mHealth apps.

There is a large diversity in the health domains considered. The mental health domain was most often addressed by the eHealth technology. The most frequent aims of the eHealth were disease-specific interventions (weight loss, psychosocial care, and rehabilitation) and self-management. The prevalence of self-management aims could be expected because the PD democratic principle emphasizes the involvement of users, and this may help the later uptake by these users of eHealth focused on self-management.

In addition to the health and eHealth technology characteristics, the theoretical references of PD are presented here. Nearly all studies, 65, mentioned a theory of PD. Clemensen et al’s description of the PD methodology [28,29] was referenced in 10 of the reviewed studies [30-39], and that by Sanders and Stappers [13] was referenced in 9 [27,30,31,40-45]. A handbook on PD by Simonsen et al [10] was referenced 7 times [27,31,36,46-49]. PD principles and practices [50,51] were also referenced on several occasions [38,47,52-54]. In addition, the methodology by Spinuzzi [2] was referenced in 4 papers [25,26,31,32]. References to other design theories were also used, such as experience-based design [55] in studies by Wherton et al [39] and Crosby [56], design thinking [57,58] in various studies [37,56,59-61], human-centered design [62] in the study by Das and Svanæs [63], and prototyping [64] in the study by Hetrick et al [65].

### Reporting on Stakeholders, Tools, and Outcome Measures

The reporting on stakeholder recruitment, stakeholder management, PD tools (make, tell, or enact), and outcomes measures to evaluate eHealth and the PD process is presented in Multimedia Appendix 3. The amount of reporting varied widely between 8 and 36 on a reporting scale of 40. All studies naturally reported on some kind of PD tools being used as this was an inclusion criterion.

Overall, 25 of the studies stated that an aim of the study was to describe the PD process or provide details of the PD process or of a design process similar to it (see gray-shaded rows in Multimedia Appendix 3). These studies scored highest on the reporting scale, with 13 of the 17 studies scoring above 30 stating that describing the PD process was an aim.

Overall, 38 studies reported on stakeholder recruitment and 30 studies reported on stakeholder management. In addition, 23 studies reported outcome measures to evaluate the eHealth technology under development, and 3 studies reported outcomes to evaluate a PD process that was already employed.

### Stakeholders

#### Types of Stakeholders

Overall, the number of participants taking part in the PD activities varied across studies. The number depended on the different types of stakeholders and the timing of the PD activities.

A total of 63 studies reported on the stakeholders involved. All of these studies involved the main intended user of the eHealth technology in the design process: the patient, the care professional, or both (see Multimedia Appendix 4 and Table 2). Among the patient, or content expert, stakeholder group, young adults and children were involved in 17 studies. Many other stakeholder types were also involved in some studies. For instance, dieticians, psychologists, a social worker, and a journalist were all involved in 1 study [46], 1 study involved a business analyst [38], 1 study a pharmacist [66], and another involved government representatives [35]. In all, 3 studies also involved, alongside a core group of stakeholders, advisory groups to provide feedback at different times [25,67,68].

**Table 2:** Types of stakeholders included in the participatory design process (n=69).

Stakeholder	Studies
Patient or content expert	[25-27,30-35,37-40,42-46,49,52-54,56,59-61,63,65,68-93]
Care professional	[26,27,31,32,34,35,37,39,41,45,46,48,56,60,61,63,65,67,68,71,73,74,76,84,87,94-99]
Informal caregiver (ie, parent)	[32,35,46,60,65,68,78,87,97]
Designer	[25,26,42,46,52,65,71,76,98]
Software developer	[25-27,38,39,42,46,48,61,63,68,74,97]
Researcher	[25-27,32,37,41,42,52,54,61,63,65,68,98]

#### Stakeholder Recruitment

The reporting on recruitment was mostly about the patient or content experts and not the other stakeholders. For instance, no study clearly explained how they recruited designers or software developers. This may be because these stakeholders were not recruited but already part of the project team. The most common recruitment strategy was purposive or convenience sampling [30,31,33,37,42,46,52,63,70,71,80,83] followed by snowball or in-person recruitment [40,65,71,79]. One study used representative sampling to include all potential users [67]. In all, 5 studies aimed for diversity in recruitment [25,45,48,81,85].

Most studies that report recruitment criteria focused on age and health care exposure. A total of 7 studies also mentioned access to internet and basic knowledge in using phones or a computer and the internet. Overall, 4 studies also reported criteria related to personal traits such as social or communicative skills, creativity, motivation, and capabilities to engage actively [31,48,85,90]. Financial incentives were also often used in the recruitment process.

In general, there is a lack of methodological arguments provided for the recruitment choices. It is unclear why designers are involved in so few studies. The PD projects may have worked with researchers who were trained in design, or they may have consulted designers before or after the PD project. Furthermore, methodological argumentation is missing on how the recruitment criteria serve the PD process and PD design aims. For instance, arguments referring to PD principles could be used to substantiate the criteria chosen. As an example, the decision to use personal trait criteria could be substantiated by stating that people who are more communicative and motivated may share more relevant knowledge than others and help others to learn from each other. These arguments could refer to the PD principle of mutual learning. Optimizing mutual learning may be particularly relevant in a health care context, given health care professionals’ limited available time.

#### Stakeholder Management

In terms of stakeholder management, creating a safe environment is important. Many approaches were reported, for example, a safe environment was sometimes fostered by creating small groups [37,63]. Sessions were deliberately shortened to reduce the burden on chronically ill patients and to give them time to reflect between sessions [49,91]. On other occasions, reassurance was provided by a researcher that no judgement was involved to avoid intimidation [40], or an explanation was provided that there was a flat communication structure [27,63].

Others mentioned the use of an icebreaker [80]. Introductions were given and sometimes also refreshments [85]. Games were used to establish the aims and rules of a workshop [71]. Others used a quick design exercise as an icebreaker, especially to get the participants used to participating in design activities [32].

Moderation was also used to reduce doubt and to seek consensus [65]. Field kits [41] or graphics [31] were used to clarify and explain concepts to clinicians and developers. Some reported that training sessions had been provided [32,47,85,92]. Information was provided using popular metaphors on key data points that were important in the design of the product or service [32]. Some studies helped children by explaining the interface and what was technically feasible during the exercises [75,89,90]. The expectations regarding a creative exploration component were clearly explained to nondesigners in one study. Elsewhere, it was made clear to the participants that the focus was on creativity and that they should not reflect on implementation at that stage [91,98]. One study [91] explicitly chose not to explain the existing technologies in order to not influence the participants and constrain their ideas.

Various approaches were taken toward the mixing of groups. Some studies chose to address the power imbalance between health professionals and patients by separating stakeholders [63,65]. Others wanted to mix stakeholders to cross-fertilize perspectives in some instances but keep subgroups by type to highlight the perceptions of a stakeholder group such as caregivers [67].

Some measures were also taken to stimulate creativity when tools were being used. To stimulate intuitive representations [32], participants were given blank cards and were invited to write on them directly [98]. Some facilitators also took an active role in helping participants suggest creative ideas but without trying to be dominant [80]. Another measure that was taken at the end of a PD session was to invite participants to walk around and look at the creations of other teams (world cafés) to increase the diversity of perspectives [32,93]. Consensus over a range of created ideas was moderated by inviting teams to evaluate the differences between ideas.

The reported facilitation varied between involving researchers and designers [42], a team of clinicians and designers [71], or a clinician and researchers [44]. Facilitation was intended to support creativity and hands-on exercises [37,48]. A mental health professional was also present during a workshop with participants who were at risk of psychological distress [73].

On some occasions, arguments related to PD principles are provided to substantiate the stakeholder management. For instance, when justifying exercises that are meant to stimulate creativity. However, further argumentation could have been provided about the relationship between creativity and the design goals.

### Tools

A variety of PD tools are used in the studies that report the development of eHealth in the predesign, early design, and post first prototype phases (see Table 3). Looking at all three phases, most combinations of tools are used in the predesign phase [31,37,39,44,97]. In this phase, 4 studies used combinations of three different types of PD tools (make, tell, and enact) [46,49,89,90]. The predesign phase is also characterized by mainly make tools that adopt a generative approach. Some studies also used a toolkit or field kit [41,47], which indicates the emphasis on helping people generate new ideas. This is different from the early design, and post first prototype phase, where fewer tools and fewer combinations of tools are used.

In all, 8 studies referred to specific techniques for a participatory prototyping approach such as *thinking aloud* [42,46,52,65,70,98], and 1 study referred to a card sorting technique for tell tools (Collaborative Analysis of Requirements and Design; CARD) [63]. Furthermore, methodological references were made to Design studio [65], Scaffold [41], the *good enough* model [71], and future workshops [80,91].

**Table 3:** Tools (n=69).

Phase and tools		Studies
**Predesign**		
	2D mapping, brainstorm, post-it, mind map, Chinese portrait [26]	[30,34,37,41,44-46,49,61,66,77,80,83-85,88,91,92,97-100]
	Prototyping, 2D mockup, 2D design, sketch	[30,31,34,37,40,42-44,46,49,61,67,77,78,89-92,98,100,101]
	Personas	[37,49,71,88]
	Cards	[31,37,39,47,49,67,84,100]
	Artifact for discussion	[85]
	Storyboarding	[31,37,39,46]
	Scenarios, customer journey	[44,66,89,90]
	Service blueprint	[66]
	Role-play	[46,49,82,89,97]
	Design journal notebook	[91]
**Early design**		
	2D mapping	[63,69]
	2D mockups, sketch	[25,32,65,68-70,72-74]
	Cards	[32,63]
	Storyboarding	[26]
	Scenarios	[56]
**Post first prototype**		
	2D mapping, brainstorm, post-its	[36,52,59,76,79,81,93,96,102]
	Prototyping, 2D or 3D mockup, sketch	[27,33,35,36,38,48,52-54,59,71,75,76,81,86,96]
	Persona	[35,79,93]
	Cards	[79,86]
	Storyboarding	[53,76]
	Scenarios, user journey	[35,54,81,93,102]
	Role-play	[79]

When looking at the substantiation offered for the PD tools used, different types of methodological arguments can be identified. Most studies argued that their main goal was to gather information or to develop, organize, or test new ideas to improve the product or service design (type 1). In many studies, an argument based on analogy is used to explain why they chose certain tools by referring to other PD literature where similar tools were used with similar design process aims (type 2).

Some authors specifically argued why they used certain generative tools by explaining the type of knowledge that they seek to capture (Type 3). Phillips et al [88] explained why they used empathy maps with people living with HIV was precisely because it is a good tool for exploring topics people feel shameful about. Ahmed et al [32] specifically highlighted their aim of using PD to visualize information in an actionable way. Some visualization tools, such as a timeline, were specifically used to capture hopes and beliefs about the future [59]. How et al stressed that their aim with PD was to merge different domains of knowledge brought together in the co-design process in their project [29]. In doing so, “the ‘Technology Domain’ comprises of selected emergent technologies that could inspire new design ideas, and the ‘Health-care Domain’ comprises of health areas that are of interest for developing new technological applications.” The authors explained that the co-design tools were specifically chosen to bring these knowledge domains together and develop a solution in this knowledge-sharing process. One study also referred to the use of certain tools including storyboards to help stakeholders express their deeper tacit knowledge [31]. In all, 4 studies [30,41,69,91] used specific generative tools such as field kits, workbooks, and design journals without explicitly reporting why these specific tools were chosen. As implied by Peters et al [30], one might assume that they were used to sensitize in the sense that they can help stakeholders express their deeper or tacit knowledge.

Some studies also related the knowledge advantage of using tools to the stakeholders involved in the PD project (type 4). This type of study justifies identifying knowledge domains related to stakeholders and then choosing outcome measures to capture that knowledge. One study explicitly stated the value of having a design expert in the teams to help select appropriate tools [37]. Another study referred to PD principles in involving clinicians as nondesigners in the design decision-making process to enhance their views and facilitate insights of others in the design [75]. This suggests that the authors related their recruitment strategy and stakeholder management to the use of PD activities and tools.

### Outcome Measures

Some of the studies evaluated the eHealth product or service output after the PD activities were concluded. The eHealth output varies depending on whether the development is in the predesign, early design, or post first prototype stage. Overall, 50 studies considered that the outputs of the PD process were in agreement with findings from similar studies or, in the case of an eHealth product, that after testing, they were effective. For instance, in an early design study, it was reported that “our design considerations show agreement with previous work related to human-factors for telerehabilitation technologies” [41]. A study where eHealth technology had been developed to a later stage reported that “we constructed an EHR-tethered PHR module named MyHealthKeeper and implemented this software in an EHR-friendly hospital” [74], which can be seen as indicating that the technology output was considered effective. Only 1 study [102] reported a negative experience: an app that had been developed for nurses did not improve the workflow, although important lessons were drawn.

Of these 50 studies that considered the outputs to be positive or effective, 22 studies reported outcome measures. These outcome measures concerned the development of the eHealth (ie, ideas developed), the quality of the eHealth (ie, usability), and the outcomes for the user (eg, body weight, managing medication, or education on health topics; see Table 4). Most of the reported outcome measures were related to usability and user feedback. As an outcome of the idea generation process, 2 studies measured the number of ideas [90,96]. Another measured the quality of new ideas: they were grouped under labels and then rated by clinicians [41]. 2 studies reported outcome measures based on clinical parameters and participation in activities for care transitioning, managing medication and education on topics such as health insurance [59,74]. There was another study reporting clinical outcome measures (not reported in Table 4); however, the authors did not make it clear whether they considered the eHealth to be effective [100].

In terms of substantiating the choices for certain outcome measures for evaluating eHealth, methodological arguments were generally missing. However, the outcome measures that How et al [41] used, such as idea grouping and the use of labels, suggest that their intention was to evaluate the knowledge development process. This could have been further substantiated by referring to PD principles related to the principles of mutual learning or creativity, for instance, to measure the impact of tools on ideas developed or shared.

Next to evaluating eHealth technology, some studies also evaluated the development process itself. Overall, 55 studies, based on the experience of the authors, considered the PD method to have successfully contributed to the eHealth development. For instance [41]:

Through a mediated exploration with clinicians and technology co-designers, we could broadly explore opportunity areas for new technologies within a healthcare domain and unravel initial design considerations related to this intersection.

Of these 55 studies that considered the method to have effectively contributed to the eHealth development, 3 studies reported outcome measures [41,45,93] (see Table 4). Outcome measures were reported regarding the quality of the knowledge development process (ie, unique ideas) and stakeholder management (ie, voices heard [45]).

When it came to substantiating the outcome measures chosen for method evaluation, methodological argumentation was again generally missing. However, the outcome measures that How et al [41] used do suggest that the intention was to evaluate the knowledge development process. The authors measured how stakeholders rated the extent to which they had an understanding of the new technology and the extent to which the use of clinical knowledge was enabled in the co-design process. Similar arguments related to knowledge expression may have driven the choice of stakeholder management outcome measures made by Revenas et al [45].

**Table 4:** Outcome measures used when electronic health technology and the participatory design method were positively evaluated (n=69).

Outcomes measures		Studies
**eHealth^a^ evaluation**		
	eHealth development (number of ideas for development)	[41,90,96]
	eHealth quality (usability, feasibility)	[30,35,46,52,53,56,63,66,68,69,71,72,75,84,90,92,96]
	User outcome (effectiveness)	[59,74]
**Participatory design method evaluation**		
	Quality of ideas (ie, unique ideas)	[41]
	Understanding of new technology through co-design process	[41]
	Enablement of clinical knowledge through co-design process	[41]
	Overall experience	[45,93]
	Workshop content in line with the aim	[45]
	Voices heard (perception)	[45]
	Balance between voiced patients and care professionals	[45]

^a^eHealth: electronic health.

## Discussion

### Principal Findings

Overall, reporting on PD methods varied significantly in studies where PD is used to develop eHealth. The extent of the reporting depended on whether or not the aim of the study was to report on the PD process itself. When it came to substantiating the methodological choices made, the justification for the tools used tended to be given the most attention.

Only a few authors explicitly used arguments directly related to PD guiding principles such as democratic, mutual learning, tacit and latent knowledge, and collective creativity. Even though the PD principles were not explicitly discussed in the method of many studies, they were implicitly identified in some. The arguments used to substantiate the choices made in stakeholder management, PD tools, and the type of outcome measures point to these principles being considered. In this discussion, the results regarding the stakeholders, tools, and outcomes are discussed separately and considered alongside other literature.

A few studies had a clear recruitment strategy, and two studies aimed for diversity in recruitment. Purposive and convenience sampling were most often used. Some studies, when reporting on recruitment, gave the recruitment strategy or the recruitment criteria. However, it was often unclear why certain stakeholders were included or excluded or why certain recruitment criteria were used. For instance, in line with the mutual learning and creativity principle, it could be expected that the recruitment strategy would aim to include designers, and this was rarely the case.

The recruitment criteria that were mentioned included age, health care exposure, access to internet, knowledge of using phones and internet, communicative skills, motivation, and capabilities to engage actively. Few studies included criteria related to personal characteristics such as communication, motivation, and engagement. This is perhaps surprising given the importance of knowledge transmission in relation to the principles of mutual learning and collective creativity. Furthermore, some studies used financial incentives to recruit individuals.

In the PD literature, the levels of expertise, passion, and creativity are suggested to play important roles in the PD process [13]. Expertise has also been suggested by others as an important condition in enhancing the creative process [103]. A meta-analysis of the PD of serious games also mentioned expertise being included as a factor of interest, but it was not found in the included studies [21]. Diversity has also been stated to play an important role in the creativity process [3,104]. Considering these personal characteristics as a whole, diversity was only identified in the recruitment strategy of a few studies in this review. This is surprising, and we would have expected the assessment of personal traits to be more prominent in the recruitment strategies in the studies included in this review.

In terms of stakeholder management, the results of this study show that various actions were taken. Moderation was aimed at providing a safe environment for equal participation, and facilitation was adopted to enhance knowledge sharing between stakeholders and to enhance creativity. This shows that some studies did consider the democratic and creativity principles of PD. Consideration was given to managing the PD process by providing a presentation about its content. In line with the principles of mutual learning and collective creativity, it may be important to manage explanations, given the different levels of expertise of care professionals, software developers, and patients involved. Overall, we had the sense that there was an implicit emphasis on creativity and understanding in some studies, but it remained unclear why a certain form of stakeholder management was chosen.

As noted above, one study may have considered the cognitive abilities of the users involved. This was also suggested in a recent meta-analysis of PD used to develop serious games where it was stressed that one should facilitate the PD tools according to the users’ cognitive abilities to increase the quality of idea generation [21]. In addition, others have also stressed that creativity can be managed on an individual level or on a group level [3,105]. Overall, it seems that adequate attention is being given to facilitating the creative process. On a personal level, creativity is correlated with a mental state of flow, and therefore, facilitating this state may play an important role in developing high-quality ideas in the PD process [16,103].

Various combinations of tools are used across the various design phases of eHealth. Some studies also described the use of toolkits, the scaffold method, the CARD technique, and the think aloud technique. The greater use of combinations of tools with a generative approach in the predesign phase may indicate that authors used these combinations to generate more new ideas. This is in line with the principles of collective creativity and tacit and latent knowledge. When looking at the arguments used to select tools, the argumentation could be categorized into four types of arguments related to knowledge development: (1) tools are used to harvest ideas for the product or service development, (2) arguments in favor of the tools based on other literature, (3) arguments explaining the aim of the tools to retrieve specific type of knowledge, and (4) arguments explaining the aim of the tools in relation to the stakeholders involved.

This focus on knowledge arguments was expected as this is implied in other publications. However, it has not yet been explicitly summarized in terms of levels of argumentation. Others have stated the importance of recognizing the fundamental role of knowledge development in PD. Given the nature of PD, this implies gaining an understanding and a generative creativity that leads in itself to different ways of knowing [4]. In terms of epistemology, the field of knowledge development is closely related to creative processes. Sanders and Stappers [3] have hinted at using social creativity theory and a *path of expression*. Even though the knowledge development theory could be a building block in a methodological framework, it is remote from practical methodological guidelines on selecting between PD tools.

In terms of outcome measures, only a limited number of studies reported outcome measures to evaluate eHealth development and the use of the PD process itself. One study in this review described the outcome measures in considerable depth for the evaluation of both eHealth and the method [41]. Compared with the other studies reviewed, this study had a more rigorous methodological framework, which also substantiated the chosen tools. This study explicitly explained that the focus was on the development of ideas and the use of different fields of expertise and knowledge. It also hinted at considerations related to knowledge developments related to the chosen tools. Nevertheless, it remains challenging to propose appropriate outcome measures to capture the output of creativity given our current understanding of it. These methodological challenges may prevent reporting the use of certain epistemological argumentations.

The identified lack of outcome measures is in line with findings elsewhere. Previous systematic reviews have also highlighted the lack of transparency about the evaluations of PD [7,8]. However, depending on the methodology and design phase, different outcome measures are suggested to evaluate the method [15,106]. Three output domains have been suggested related to the stakeholders (ie, empowerment), to knowledge (ie, tacit, pragmatic, and technical), and to implementation (ie, ownership) [16].

### Limitations

The results of this study are limited for several reasons. First, the search strategy for relevant research is limited by the focus on papers published in scientific journals. Given that many reports on PD in developing eHealth are not in scientific journals, the review only provides a partial view of the state of reporting PD methodology, namely only that in the empirical scientific literature.

The screening process is limited by the definitions applied for the terms used in the inclusion and exclusion criteria. As there is no universally agreed definition of PD, a working definition was chosen that focuses on one strand of PD research, namely where stakeholders are a partner in the process. Consequently, studies describing PD in a more user-centered way were excluded, and their inclusion may have led to different results.

Turning to the analysis and conclusions, the following limitations were identified. First, it is challenging to draw conclusions based on the reporting of the PD methods as described in the papers selected in the systematic literature review. The actual methodological intentions and considerations made during the PD project may differ from what is reported in the studies. The limited number of studies reporting outputs and outcome measures may be related to the recognized publication bias toward reporting positive results and eHealth products and services that are already fairly developed. In addition, the evaluation of the eHealth technology may have been reported in a separate publication; for example, in the paper by Waller et al [98] included in this review, it is noted that the results of the randomized controlled trial of the eHealth technology are reported elsewhere [107], and the latter paper did not meet the inclusion criteria for this review. This was because studies that focused on the outcome measures of the eHealth technology were excluded from this review.

### Implications

The PD methodology is still under development [2,4,108]. Providing methodological reasoning in a transparent way about the choices of stakeholders, tools, and outcome measures employed is important for methodological progress. A clear PD methodology could well enhance the development of eHealth in practice as practitioners would then be able to argue more rigorously for a certain form of PD. A clear methodology may also improve the rigor and accountability of the science of PD. For instance, given a methodology, evaluation criteria could be used to evaluate the method, which can then inform other researchers about how it can be further improved. A clear methodology may also help to select an appropriate form of PD for a specific research design.

### Reflection

The fact that the methodological reasonings behind the use of PD are not widely reported could be because of several reasons. From a scientific perspective, PD has mixed origins, ranging from social science through action research to the design sciences [3]. This may result in different scientific reporting styles appearing across the scientific literature; for example, the theoretical underpinnings of a methodology tend to be much less described in empirical literature in the health sciences than elsewhere.

The academic design culture is still developing alongside other different cultures such as engineering, the arts, and the social sciences [109]. Although classical research methods and design methods are closely related, they are different. In the PD science field, one sees many different crossovers; for example, one can involve research for a single aspect during a design project but also fully incorporate research methods at every design step. Depending on how research is used in a PD project, the reporting will differ. When the emphasis is on scientific reporting, the methodological steps tend to be explained, but when the emphasis is on design reporting, the design products will be more heavily emphasized. Looking at the results of our study from this perspective, one could argue that the majority of the authors have put the emphasis on design reporting and less on scientific reporting.

This observation can be further explained using the observations by Spinuzzi [2], who claimed that there is no strong methodological justification for PD in the first place. Although there are some principles, stated in this study, on how PD should be conducted, a methodological framework for PD is scarcely discussed [2,4]. This may leave researchers confused as to how to employ and report on PD methodology.

PD reporting could be improved if PD researchers were to adopt a more *scientific* attitude toward carrying out a PD project. Improving documentation on the choices of certain PD recruitment strategies, the use of certain tools, or the use of outcome measures could provide more information that could then be reported in scientific journals. Improving education about the scientific documentation of PD projects for designers and eHealth developers could help to improve future reporting. One key challenge here is to translate design terminology to scientific terminology and vice versa; for example, prototype testing in design might be translated as hypothesis testing in science.

### Further Research

Further research can help improve the methodological framework for PD in eHealth. A particular focus on the knowledge development process, as a core aspect of PD, would greatly help in substantiating methodological choices and in measuring the outputs of a PD process, especially in eHealth given the various areas of technical knowledge involved. There is a growing interest in the methodology of design known as *Research through Design* [109], which could help foster the development of a methodological framework for PD that would help develop better eHealth.

### Conclusions

Studies that use a PD research methodology to develop eHealth primarily substantiate the choice of tools and much less the selection of stakeholders and outcome measures.

## Appendix

Multimedia Appendix 1 Systematic review: search strategy.

Multimedia Appendix 2 Excluded studies.

Multimedia Appendix 3 Reporting on stakeholders, tools and outcomes.

Multimedia Appendix 4 Stakeholder recruitment and management.

## Abbreviations

CARD: Collaborative Analysis of Requirements and Design
eHealth: electronic health
mHealth: mobile health
PD: participatory design
PRISMA: Preferred Reporting Items for Systematic Reviews and Meta-Analyses
