# Supplementary material for: Generative Participatory Design Methodology to Develop Electronic Health Interventions: Systematic Literature Review
Source: J Med Internet Res. 2020 Apr 27;22(4):e13780. doi: 10.2196/13780 (PMC7215492; doi:10.2196/13780)
Supplement: Multimedia Appendix 1 [file jmir_v22i4e13780_app1.docx]

**Multimedia Appendix 1: Search strategy**

| **Database** | **Hits** | **Hits (without duplicates)** |
| --- | --- | --- |
| Embase.com | 1551 | 1525 |
| Medline Ovid | 1095 | 287 |
| Web of science | 483 | 144 |
| CINAHL EBSCOhost | 1527 | 1175 |
| **Total** | **4656** | **3131** |

**Embase.com**

((cocreat* OR co-creat* OR co-design* OR codesign* OR ((particip* OR collaborat* OR cooperat* OR engag*) NEXT/1 (design*))):ab,ti) OR (((((particip* OR collaborat* OR cooperat* OR engag* OR human-cent* OR user-cent* OR user-involv* OR patient*-cent* OR patient*-involv* OR patient*-partner* OR patient*-activat* OR partner* OR iterat*) NEAR/3 (predesign* OR design* OR produc* OR sales OR market* OR problem*-solv* OR solution* OR front-end OR frontend)) OR co-produc* OR coproduc*):ab,ti) AND ('doctor patient relation'/de OR 'shared decision making'/de OR (((doctor* OR clinician* OR technologist* OR staff* OR practitioner* OR professional* OR designer* OR engineer*) NEAR/6 (carer* OR caregiver* OR patient* OR people-with OR family*)) OR (shar* NEAR/3 decision*)):ab,ti)) AND ('telehealth'/exp OR 'mobile application'/de OR 'mobile phone'/exp OR internet/exp OR 'social media'/de OR multimedia/de OR 'computer'/exp OR 'computer interface'/de OR 'e learning'/de OR 'electronic learning'/de OR 'big data'/de OR 'electronic health record'/de OR 'electronic medical record'/de OR 'information and communication technology'/de OR 'information technology'/de OR (telehealth OR tele-health OR ehealth OR e-health OR teletherap* OR telesurger* OR telerehabilitat* OR teleradiotherap* OR teleradiolog* OR telepsychiatr* OR telepatholog* OR telemonitor* OR telemedicine* OR telediagnos* OR teledermatolog* OR teleconsult* OR telecardiolog* OR telenursing* OR tele-therap* OR tele-surger* OR tele-rehabilitat* OR tele-radiotherap* OR tele-radiolog* OR tele-psychiatr* OR tele-patholog* OR tele-monitor* OR tele-medicine* OR tele-diagnos* OR tele-dermatolog* OR tele-consult* OR tele-cardiolog* OR tele-nursing* OR mhealth OR m-health OR app OR ((mobile OR interactive* OR digital*) NEAR/3 (application* OR technolog* OR device* OR tool*)) OR smartphone* OR cellphone* OR ((smart OR cell OR cellular* OR mobile) NEXT/1 (phone*)) OR webbased OR web-based OR www OR world-wide-web OR website* OR web-site* OR online OR on-line OR internet OR multimedia OR YouTube* OR 'social media' OR facebook OR twitter OR multimedia OR computer* OR minicomputer* OR wiki OR elearning OR e-learning OR electronic-learning OR big-data OR (electronic* NEAR/6 (health OR medical*) NEAR/3 record*) OR ehr OR (information* NEAR/3 technolog*) OR ict OR (patient* NEAR/3 portal*)):ab,ti) AND [english]/lim NOT ([animals]/lim NOT [humans]/lim)

**Medline Ovid**

((cocreat* OR co-creat* OR co-design* OR codesign* OR ((particip* OR collaborat* OR cooperat* OR engag*) ADJ (design*))).ab,ti.) OR (((((particip* OR collaborat* OR cooperat* OR engag* OR human-cent* OR user-cent* OR user-involv* OR patient*-cent* OR patient*-involv* OR patient*-partner* OR patient*-activat* OR partner* OR iterat*) ADJ3 (predesign* OR design* OR produc* OR sales OR market* OR problem*-solv* OR solution* OR front-end OR frontend)) OR co-produc* OR coproduc*).ab,ti.) AND (Physician-Patient Relations/ OR (((doctor* OR clinician* OR technologist* OR staff* OR practitioner* OR professional* OR designer* OR engineer*) ADJ6 (carer* OR caregiver* OR patient* OR people-with OR family*)) OR (shar* ADJ3 decision*)).ab,ti.)) AND (exp Telemedicine/ OR Mobile Applications/ OR exp Cell Phone/ OR Internet/ OR Social Media/ OR Multimedia/ OR exp Computers/ OR User-Computer Interface/ OR Electronic Health Records/ OR Information Technology/ OR (telehealth OR tele-health OR ehealth OR e-health OR teletherap* OR telesurger* OR telerehabilitat* OR teleradiotherap* OR teleradiolog* OR telepsychiatr* OR telepatholog* OR telemonitor* OR telemedicine* OR telediagnos* OR teledermatolog* OR teleconsult* OR telecardiolog* OR telenursing* OR tele-therap* OR tele-surger* OR tele-rehabilitat* OR tele-radiotherap* OR tele-radiolog* OR tele-psychiatr* OR tele-patholog* OR tele-monitor* OR tele-medicine* OR tele-diagnos* OR tele-dermatolog* OR tele-consult* OR tele-cardiolog* OR tele-nursing* OR mhealth OR m-health OR app OR ((mobile OR interactive* OR digital*) ADJ3 (application* OR technolog* OR device* OR tool*)) OR smartphone* OR cellphone* OR ((smart OR cell OR cellular* OR mobile) ADJ (phone*)) OR webbased OR web-based OR www OR world-wide-web OR website* OR web-site* OR online OR on-line OR internet OR multimedia OR YouTube* OR social media OR facebook OR twitter OR multimedia OR computer* OR minicomputer* OR wiki OR elearning OR e-learning OR electronic-learning OR big-data OR (electronic* ADJ6 (health OR medical*) ADJ3 record*) OR ehr OR (information* ADJ3 technolog*) OR ict OR (patient* ADJ3 portal*)).ab,ti.) AND english.la. NOT (exp animals/ NOT humans/)

**Web of science**

TS=((((((cocreat* OR co-creat* OR co-design* OR codesign* OR ((particip* OR collaborat* OR cooperat* OR engag*) NEAR/1 (design*)))) OR ((particip* OR collaborat* OR cooperat* OR engag* OR human-cent* OR user-cent* OR user-involv* OR patient*-cent* OR patient*-involv* OR patient*-partner* OR patient*-activat* OR partner* OR iterat*) NEAR/2 (predesign* OR design* OR produc* OR sales OR market* OR problem*-solv* OR solution* OR front-end OR frontend)) OR co-produc* OR coproduc*)) AND ((((doctor* OR clinician* OR technologist* OR staff* OR practitioner* OR professional* OR designer* OR engineer*) NEAR/5 (carer* OR caregiver* OR patient* OR people-with OR family*)) OR (shar* NEAR/2 decision*)))) AND ((telehealth OR tele-health OR ehealth OR e-health OR teletherap* OR telesurger* OR telerehabilitat* OR teleradiotherap* OR teleradiolog* OR telepsychiatr* OR telepatholog* OR telemonitor* OR telemedicine* OR telediagnos* OR teledermatolog* OR teleconsult* OR telecardiolog* OR telenursing* OR tele-therap* OR tele-surger* OR tele-rehabilitat* OR tele-radiotherap* OR tele-radiolog* OR tele-psychiatr* OR tele-patholog* OR tele-monitor* OR tele-medicine* OR tele-diagnos* OR tele-dermatolog* OR tele-consult* OR tele-cardiolog* OR tele-nursing* OR mhealth OR m-health OR app OR ((mobile OR interactive* OR digital*) NEAR/2 (application* OR technolog* OR device* OR tool*)) OR smartphone* OR cellphone* OR ((smart OR cell OR cellular* OR mobile) NEAR/1 (phone*)) OR webbased OR web-based OR www OR world-wide-web OR website* OR web-site* OR online OR on-line OR internet OR multimedia OR YouTube* OR "social media" OR facebook OR twitter OR multimedia OR computer* OR minicomputer* OR wiki OR elearning OR e-learning OR electronic-learning OR big-data OR (electronic* NEAR/5 (health OR medical*) NEAR/2 record*) OR ehr OR (information* NEAR/2 technolog*) OR ict OR (patient* NEAR/2 portal*)))) AND LA=(english)

**CINAHL EBSCOhost**

(TI(cocreat* OR co-creat* OR co-design* OR codesign*) OR AB (cocreat* OR co-creat* OR co-design* OR codesign*)) OR ((TI (((particip* OR collaborat* OR cooperat* OR engag* OR human-cent* OR user-cent* OR user-involv* OR patient*-cent* OR patient*-involv* OR patient*-partner* OR patient*-activat* OR partner* OR iterat*) N2 (predesign* OR design* OR produc* OR sales OR market* OR problem*-solv* OR solution* OR front-end OR frontend)) OR co-produc* OR coproduc*) OR AB (((particip* OR collaborat* OR cooperat* OR engag* OR human-cent* OR user-cent* OR user-involv* OR patient*-cent* OR patient*-involv* OR patient*-partner* OR patient*-activat* OR partner* OR iterat*) N2 (predesign* OR design* OR produc* OR sales OR market* OR problem*-solv* OR solution* OR front-end OR frontend)) OR co-produc* OR coproduc*)) AND (TI(((doctor* OR clinician* OR technologist* OR staff* OR practitioner* OR professional* OR designer* OR engineer*) N5 (carer* OR caregiver* OR patient* OR people-with OR family*)) OR (shar* N2 decision*) OR AB (((doctor* OR clinician* OR technologist* OR staff* OR practitioner* OR professional* OR designer* OR engineer*) N5 (carer* OR caregiver* OR patient* OR people-with OR family*)) OR (shar* N2 decision*))))) AND (MH Telemedicine+ OR MH Mobile Applications OR MH Cellular Phone+ OR MH Internet OR MH Social Media OR MH Multimedia OR MH "Computers and Computerization+" OR MH User-Computer Interface OR MH Electronic Health Records OR MH Information Technology OR TI (telehealth OR tele-health OR ehealth OR e-health OR teletherap* OR telesurger* OR telerehabilitat* OR teleradiotherap* OR teleradiolog* OR telepsychiatr* OR telepatholog* OR telemonitor* OR telemedicine* OR telediagnos* OR teledermatolog* OR teleconsult* OR telecardiolog* OR telenursing* OR tele-therap* OR tele-surger* OR tele-rehabilitat* OR tele-radiotherap* OR tele-radiolog* OR tele-psychiatr* OR tele-patholog* OR tele-monitor* OR tele-medicine* OR tele-diagnos* OR tele-dermatolog* OR tele-consult* OR tele-cardiolog* OR tele-nursing* OR mhealth OR m-health OR app OR ((mobile OR interactive* OR digital*) N2 (application* OR technolog* OR device* OR tool*)) OR smartphone* OR cellphone* OR ((smart OR cell OR cellular* OR mobile) N1 (phone*)) OR webbased OR web-based OR www OR world-wide-web OR website* OR web-site* OR online OR on-line OR internet OR multimedia OR YouTube* OR social media OR facebook OR twitter OR multimedia OR computer* OR minicomputer* OR wiki OR elearning OR e-learning OR electronic-learning OR big-data OR (electronic* N5 (health OR medical*) N2 record*) OR ehr OR (information* N2 technolog*) OR ict OR (patient* N2 portal*)) OR AB (telehealth OR tele-health OR ehealth OR e-health OR teletherap* OR telesurger* OR telerehabilitat* OR teleradiotherap* OR teleradiolog* OR telepsychiatr* OR telepatholog* OR telemonitor* OR telemedicine* OR telediagnos* OR teledermatolog* OR teleconsult* OR telecardiolog* OR telenursing* OR tele-therap* OR tele-surger* OR tele-rehabilitat* OR tele-radiotherap* OR tele-radiolog* OR tele-psychiatr* OR tele-patholog* OR tele-monitor* OR tele-medicine* OR tele-diagnos* OR tele-dermatolog* OR tele-consult* OR tele-cardiolog* OR tele-nursing* OR mhealth OR m-health OR app OR ((mobile OR interactive* OR digital*) N2 (application* OR technolog* OR device* OR tool*)) OR smartphone* OR cellphone* OR ((smart OR cell OR cellular* OR mobile) N1 (phone*)) OR webbased OR web-based OR www OR world-wide-web OR website* OR web-site* OR online OR on-line OR internet OR multimedia OR YouTube* OR social media OR facebook OR twitter OR multimedia OR computer* OR minicomputer* OR wiki OR elearning OR e-learning OR electronic-learning OR big-data OR (electronic* N5 (health OR medical*) N2 record*) OR ehr OR (information* N2 technolog*) OR ict OR (patient* N2 portal*))) AND LA(english) NOT (MH animals+ NOT MH humans+)
