# Supplementary material for: Generative Participatory Design Methodology to Develop Electronic Health Interventions: Systematic Literature Review
Source: J Med Internet Res. 2020 Apr 27;22(4):e13780. doi: 10.2196/13780 (PMC7215492; doi:10.2196/13780)
Supplement: Multimedia Appendix 2 [file jmir_v22i4e13780_app2.docx]

**Multimedia Appendix 2: Excluded studies (Total 62)**

| 1 | Abbass-Dick J, Brolly M, Huizinga J, Newport A, Xie F, George S, Sterken E. Designing an eHealth Breastfeeding Resource With Indigenous Families Using a Participatory Design. J Transcult Nurs [Internet] 2018;29(5):480–488. [doi: 10.1177/1043659617731818] |
| --- | --- |
| 2 | Arevian AC, O’Hora J, Jones F, Mango J, Jones L, Williams PG, Booker-Vaughns J, Jones A, Pulido E, Banner-Jackson D, Wells KB. Participatory Technology Development to Enhance Community Resilience. Ethn Dis [Internet] 2018;28(Suppl 2):493–502. PMID:30202203 |
| 3 | Argenas A, Myers B, Witteman H, Arnold RM, Shields A, Buddadhumaruk P, Cox C, White DB. Developing a web-based tool to enhance communication and shared decision making for families of critically ill patients through user-centered methods. Am J Respir Crit Care Med [Internet] 2018;197(MeetingAbstracts). Available from: http://www.embase.com/search/results?subaction=viewrecord&from=export&id=L622965056 |
| 4 | Balli F. Developing Digital Games to Address Airway Clearance Therapy in Children With Cystic Fibrosis: Participatory Design Process. JMIR Serious Games [Internet] 2018;6(4):e18. PMID:30463835 |
| 5 | Billis A, Mantziari D, Zilidou V, Bamidis PD. Co-Creation of an Innovative Vocational Training Platform to Improve Autonomy in the Context of Alzheimer’s Disease. Stud Heal Technol Inf [Internet] 2018;251:309–312. PMID:29968665 |
| 6 | Blusi M, Nilsson I, Lindgren H. Older Adults Co-Creating Meaningful Individualized Social Activities Online for Healthy Ageing. Stud Heal Technol Inf [Internet] 2018;247:775–779. Available from: http://www.embase.com/search/results?subaction=viewrecord&from=export&id=L622785805 |
| 7 | Branch-Smith C, Shaw T, Lin A, Runions K, Payne D, Ngyuen R, Hugo H, Cross D. Developing an innovative online intervention to support schooling for children and young people with cystic fibrosis. J Cyst Fibros [Internet] 2017;16:S39. Available from: http://www.embase.com/search/results?subaction=viewrecord&from=export&id=L620749518 |
| 8 | Brown DJ, Battersby S, Standen P, Anderton N, Harrison M. Inclusive design for disability: A prototype switch for use in virtual learning environments. J Endocr Genet [Internet] 2005;4(2):103–119. Available from: http://www.embase.com/search/results?subaction=viewrecord&from=export&id=L44783456 |
| 9 | Cahill J, Portales R, McLoughin S, Nagan N, Henrichs B, Wetherall S. IoT/Sensor-Based Infrastructures Promoting a Sense of Home, Independent Living, Comfort and Wellness. Sensors (Basel) [Internet] 2019;19(3). [doi: 10.3390/s19030485] |
| 10 | Cardno EJ. Managing the “fit” of information and communication technology in community health: a framework for decision making. J Telemed Telecare 2000;6:6–8. |
| 11 | Coorey G, Neubeck L, Peiris D, Hersch F, Patel B, Lyford M, Wechsler J, Tan L, Redfern J. The use of journey mapping and persona creation to inform design of an e-health strategy to support cardiovascular disease prevention. Glo Hear [Internet] 2014;9(1):e39. [doi: 10.1016/j.gheart.2014.03.1351] |
| 12 | Daniels J, Schwartz J, Haber N, Voss C, Kline A, Fazel A, Washington P, De T, Feinstein C, Winograd T, Wall D. Design and efficacy of a wearable device for social affective learning in children with autism. J Am Acad Child Adolesc Psychiatry [Internet] 2017;56(10):S257. [doi: 10.1016/j.jaac.2017.09.296] |
| 13 | Darking M, Henwood F, Marent B, West B, Whetham J. EmERGE: Codesigning mHealth to support access to records and reduced visit pathways in patients living with stable HIV. HIV Med [Internet] 2018;19:S100–S101. Available from: http://www.embase.com/search/results?subaction=viewrecord&from=export&id=L621998964 |
| 14 | Easton K, Burton T, Ariss S, Bradburn M, Hawley M. Smart Clothing for Falls Protection and Detection: User-Centred Co-Design and Feasibility Study. Stud Heal Technol Inf [Internet] IOS Press; 2017;242:152–159. [doi: 10.3233/978-1-61499-798-6-152] |
| 15 | Ellis RD, Jankowski TB, Jasper JE. Participatory design of an internet-based information system for aging services professionals. Gerontologist [Internet] 1998;38(6):743–748. [doi: 10.1093/geront/38.6.743] |
| 16 | Elsbernd A, Hjerming M, Visler C, Hjalgrim LL, Niemann CU, Boisen KA, Jakobsen J, Pappot H. Using Cocreation in the Process of Designing a Smartphone App for Adolescents and Young Adults With Cancer: Prototype Development Study. JMIR Form Res [Internet] 2018;2(2):e23. PMID:30684439 |
| 17 | Flohr L, Beaudry S, Johnson KT, West N, Burns CM, Ansermino JM, Dumont GA, Wensley D, Skippen P, Gorges M. Clinician-Driven Design of VitalPAD-An Intelligent Monitoring and Communication Device to Improve Patient Safety in the Intensive Care Unit. IEEE J Transl Eng Heal Med [Internet] 2018;6. [doi: 10.1109/jtehm.2018.2812162] |
| 18 | Foster JM, Peters D, Davis S, Calvo R, Sawyer S, Smith L. Using a co-design approach to develop an appealing goal-setting and self-management app for young people with asthma. Am J Respir Crit Care Med [Internet] 2017;195. [doi: 10.1164/ajrccm-conference.2017.B38] |
| 19 | Frauenberger C, Spiel K, Makhaeva J. Thinking OutsideTheBox - Designing Smart Things with Autistic Children. Int J Hum Comput Interact [Internet] 2019;35(8):666–678. PMID:31057337 |
| 20 | Garne Holm K, Brødsgaard A, Zachariassen G, Smith AC, Clemensen J. Participatory design methods for the development of a clinical telehealth service for neonatal homecare. SAGE Open Med [Internet] 2017;5. [doi: 10.1177/2050312117731252] |
| 21 | Goeman D, Michael J, King J, Luu H, Emmanuel C, Koch S. Partnering with consumers to develop and evaluate a Vietnamese Dementia Talking-Book to support low health literacy: a qualitative study incorporating codesign and participatory action research. BMJ Open [Internet] 2016;6(9):e011451. PMID:27670516 |
| 22 | Hahn-Goldberg S, Damba C, Solomon R, Okrainec K, Abrams H, Tai H. Using Co-Design Methods to Create a Patient-Oriented Discharge Summary. JCOM [Internet] Wayne, Pennsylvania: Turner White Communications; 2016;23(7):321–328. Available from: https://search.ebscohost.com/login.aspx?direct=true&db=jlh&AN=116884153&site=ehost-live |
| 23 | Hamidi F, Baljko M, Ecomomopoulos C, Livingston NJ, Spalteholz LG. Co-designing a speech interface for people with dysarthria. J Assist TECHNOL [Internet] Emerald Publishing; 2015;9(3):159–173. [doi: 10.1108/JAT-10-2014-0026] |
| 24 | Hardy A, Garety P. Mo: Development and feasibility testing of a mobile therapy app for transdiagnostic early intervention. Early Interv Psychiatry [Internet] 2018;12:76. [doi: 10.1111/eip.12723] |
| 25 | Haufe M, Peek STM, Luijkx KG. Matching gerontechnologies to independent-living seniors’ individual needs: development of the GTM tool. BMC Heal Serv Res [Internet] BioMed Central; 2019;19(1):N.PAG-N.PAG. [doi: 10.1186/s12913-018-3848-5] |
| 26 | Hjelmfors L, Stromberg A, Friedrichsen M, Sandgren A, Martensson J, Jaarsma T. Using co-design to develop an intervention to improve communication about the heart failure trajectory and end-of-life care. BMC Palliat Care [Internet] 2018;17(1):85. PMID:29890974 |
| 27 | Hochstenbach LMJ, Courtens AM, Zwakhalen SMG, Vermeulen J, van Kleef M, de Witte LP. Co-creative development of an eHealth nursing intervention: Self-management support for outpatients with cancer pain. Appl Nurs Res [Internet] 2017;36:1–8. [doi: 10.1016/j.apnr.2017.03.004] |
| 28 | Johnson KT, Peters C, West N, Ansermino JM, Wensley D, Skippen P, Görges M. Vitalpad: Designing a mobile monitoring and communication application to support pediatric intensive care in a newly designed academic acute care centre. Pediatr Crit Care Med [Internet] 2018;19(6):70–71. Available from: http://www.embase.com/search/results?subaction=viewrecord&from=export&id=L623815998 |
| 29 | Kariyawasam N, Ming Chao W, Turner P. User Centred Design and Nosocomials in Surgical ICUs: A Mobile Application for Peer Monitoring and Training in Hand Hygiene...The 16 World Congress of Medical and Health Informatics: Precision Healthcare Through Informatics (MedInfo2017) was held in Hangz. Stud Heal Technol Inf [Internet] IOS Press; 2018;245:743–747. [doi: 10.3233/978-1-61499-830-3-743] |
| 30 | Kildea J, Hijal T, Hendren L. Development of a person-centered patient portal in oncology using stakeholder codesign. J Clin Oncol [Internet] 2018;36(30). [doi: 10.1200/JCO.2018.36.30-suppl.221] |
| 31 | Latulippe K, Tremblay M, Poulin V, Provencher V, Giguere AM, Sevigny A, Dube V, Ethier S, Guay M, Carignan M, Giroux D. Prioritizing the Needs of Caregivers of Older Adults to Support Their Help-Seeking Process as a First Step to Developing an eHealth Tool: The Technique for Research of Information by Animation of a Group of Experts (TRIAGE) Method. JMIR Aging [Internet] 2019;2(1):e12271. PMID:31518269 |
| 32 | Lo J, Moore J, Wedlake C, Guiraudon G, Eagleson R, Peters T. Surgeon-controlled visualization techniques for virtual reality-guided cardiac surgery. Stud Heal Technol Inf [Internet] 2009;142:162–167. Available from: https://books.google.nl/books?id=9HbAhWChR58C&pg=PA162&dq=Surgeon+controlled+visualization+techniques+for+virtual+reality+guided+cardiac+surgery&hl=en&sa=X&ved=0ahUKEwi6-7qf8_XlAhUOPFAKHabiAEAQ6AEIKzAA#v=onepage&q=Surgeon controlled visualization techniqu |
| 33 | Lundin M, Mäkitalo Å. Co-designing technologies in the context of hypertension care: Negotiating participation and technology use in design meetings. Inf Heal Soc Care [Internet] 2017;42(1):18–31. Available from: http://www.embase.com/search/results?subaction=viewrecord&from=export&id=L616183199 |
| 34 | Man J, Woolridge N, Jenkinson J, Wall S, Bartlett S, McCune M. Human-centred research and its application to the rapid and iterative design of digital treatment-decision-making support tools. Psychooncology [Internet] 2013;22:356–357. [doi: 10.10002/pon.3394] |
| 35 | Marent B, Henwood F, Darking M, Em EC. Development of an mHealth platform for HIV Care: Gathering User Perspectives Through Co-Design Workshops and Interviews. JMIR Mhealth Uhealth [Internet] 2018;6(10):e184. PMID:30339132 |
| 36 | Marko-Holguin M, Cordel SL, Van Voorhees BW, Fogel J, Sykes E, Fitzgibbon M, Glassgow AE. A Two-Way Interactive Text Messaging Application for Low-Income Patients with Chronic Medical Conditions: Design-Thinking Development Approach. JMIR Mhealth Uhealth [Internet] 2019;7(5):e11833. PMID:31042152 |
| 37 | McClelland GT, Fitzgerald M. A participatory mobile application (app) development project with mental health service users and clinicians. Heal Educ J [Internet] 2018; [doi: 10.1177/0017896918773790] |
| 38 | Moen A, Smørdal O, Sem I. Web-based resources for peer support - Opportunities and challenges. Stud Heal Technol Informatics [Internet] p. 302–306. [doi: 10.3233/978-1-60750-044-5-302] |
| 39 | Moen A, Smørdal O. RareICT: a web-based resource to augment self-care and independence with a rare medical condition. Work [Internet] 2012;41(3):329–337. Available from: http://www.embase.com/search/results?subaction=viewrecord&from=export&id=L365271621 |
| 40 | Mozzilli SL, Sassi E, Rossi L, Sonaglio V, Francisco F, Duarte A, Dos Santos LP, Yabu G, Sredni ST. Creating and developing games throughout interdisciplinarity and patient participation. J Clin Oncol [Internet] 2017;35(15). Available from: http://www.embase.com/search/results?subaction=viewrecord&from=export&id=L617537572 |
| 41 | Nasr N, Leon B, Mountain G, Nijenhuis SM, Prange G, Sale P, Amirabdollahian F. The experience of living with stroke and using technology: opportunities to engage and co-design with end users. Disabil Rehabil Assist Technol [Internet] 2016;11(8):653–660. [doi: 10.3109/17483107.2015.1036469] |
| 42 | Nguyen MH, Bol N, van Weert JCM, Loos EF, Tytgat K, Geijsen D, Drenth E, Janse M, Smets EMA. Optimising eHealth tools for older patients: Collaborative redesign of a hospital website. Eur J Cancer Care [Internet] 2018;e12882. PMID:30015998 |
| 43 | Nilsson L, Hofflander M. What if It Was Like a Departure Lounge at an Airport? - eHealth for Healthcare Staff in a Swedish Healthcare Organization, a Participatory Design Study. Stud Heal Technol Inf [Internet] 2016;225:923–924. Available from: http://www.embase.com/search/results?subaction=viewrecord&from=export&id=L621262156 |
| 44 | Nygardh A, Martensson J, Allemann H, Stromberg A, Aidemark J, Fruberg C, Karlsson JE, Askenas L. An experience-based co-design to accomplish person-centered self-care support for elderly persons with heart failure. Eur J Cardiovasc Nurs [Internet] 2015;14:51–52. [doi: 10.1177/1474515115579615] |
| 45 | Pappot H, Assam Taarnhøj G, Elsbernd A, Hjerming M, Hanghøj S, Jensen M, Boisen KA. Health-Related Quality of Life Before and After Use of a Smartphone App for Adolescents and Young Adults With Cancer: Pre-Post Interventional Study. JMIR Mhealth Uhealth [Internet] 2019;7(10):e13829. [doi: 10.2196/13829] |
| 46 | Pappot H, Hjerming M, Petersen G, Boisen KA, Niemann CU, Hjalgrim LL. Patient development of an electronic tool to empower adolescents and young adults with cancer. Eur J Cancer [Internet] 2017;72:S175–S176. Available from: http://www.embase.com/search/results?subaction=viewrecord&from=export&id=L621797058 |
| 47 | Peerbhai C, Grootoonk S, De Waal H, McLeish K, Hill DL. Co-designing a digital platform to support people with dementia and their carers. Alzheimer’s Dement [Internet] 2016;12(7):P154–P155. Available from: http://www.embase.com/search/results?subaction=viewrecord&from=export&id=L613187234 |
| 48 | Pereira RVS, Kubrusly M, Nogueira IC, Gondim VJT, Marçal E. Development of an application to support in-service training of anesthesiologists on preoperative evaluation in a public hospital in Brazil. J Eval Clin Pr [Internet] 2019;25(5):850–855. [doi: 10.1111/jep.13112] |
| 49 | Robinson L, Brittain K, Lindsay S, Jackson D, Olivier P. Keeping in Touch Everyday (KITE) project: Developing assistive technologies with people with dementia and their carers to promote independence. Int Psychogeriatr [Internet] 2009;21(3):494–502. [doi: 10.1017/s1041610209008448] |
| 50 | Rose M, Carragher M, Taylor N, Johnson H, Torabi T, O’Halloran R. The Aphasia App: A novel technology-based approach to improving healthcare communication for people with post stroke aphasia and healthcare professionals. Int J Stroke [Internet] 2017;12(3):57. [doi: 10.1177/1747493017720548] |
| 51 | Rudin RS, Fanta CH, Predmore Z, Kron K, Edelen MO, Landman AB, Zimlichman E, Bates DW. Core Components for a Clinically Integrated mHealth App for Asthma Symptom Monitoring. Appl Clin Inf [Internet] 2017;8(4):1031–1043. [doi: 10.4338/aci-2017-06-ra-0096] |
| 52 | Salman YB, Cheng HI, Patterson PE. Icon and user interface design for emergency medical information systems: A case study. Int J Med Informatics [Internet] 2012;81(1):29–35. [doi: 10.1016/j.ijmedinf.2011.08.005] |
| 53 | Stevens MCG, Beynon P, Cameron A, Cargill J, Cheshire J, Dolby S. Understanding and Utilizing the Unmet Needs of Teenagers and Young Adults with Cancer to Determine Priorities for Service Development: The Macmillan On Target Programme. 2018; PMID:29989481 |
| 54 | Timmerman JG, Tönis TM, Dekker-van Weering MG, Stuiver MM, Wouters MW, van Harten WH, Hermens HJ, Vollenbroek-Hutten MM. Co-creation of an ICT-supported cancer rehabilitation application for resected lung cancer survivors: design and evaluation. BMC Heal Serv Res [Internet] 2016;16:155. [doi: 10.1186/s12913-016-1385-7] |
| 55 | Treadaway C, Kenning G. Sensor e-textiles: person centered co-design for people with late stage dementia. Work OLDER PEOPLE [Internet] Emerald Publishing; 2016;20(2):76–85. [doi: 10.1108/WWOP-09-2015-0022] |
| 56 | van Bruinessen IR, van Weel-Baumgarten EM, Snippe HW, Gouw H, Zijlstra JM, van Dulmen S. Active patient participation in the development of an online intervention. JMIR Res Protoc [Internet] 2014;3(4):e59. PMID:25379679 |
| 57 | Van Gils RHJ, Helder OK, Wauben LSGL. Incubator traffic light: The development of an alcohol-based hand rub dispenser system for neonatal incubators with visual feedback to improve hand hygiene compliance. BMJ Innov [Internet] 2019; [doi: 10.1136/bmjinnov-2018-000301] |
| 58 | Van Velsen L, Illario M, Jansen-Kosterink S, Crola C, Di Somma C, Colao A, Vollenbroek-Hutten M. A community-based, technology-supported health service for detecting and preventing frailty among older adults: A participatory design development process. J Aging Res [Internet] 2015;2015. [doi: 10.1155/2015/216084] |
| 59 | Webb M, Wadley G, Kauer S, Sanci LA. Check Up GP: Implementing a co-designed health and lifestyle screening app to improve patient-centred care for young people in primary care. J Adolesc Heal [Internet] 2017;60(2):S14–S15. Available from: http://www.embase.com/search/results?subaction=viewrecord&from=export&id=L615030126 |
| 60 | Woods L, Cummings E, Duff J, Walker K. Partnering in Digital Health Design: Engaging the Multidisciplinary Team in a Needs Analysis. Stud Heal Technol Inf [Internet] 2018;252:176–181. PMID:30040702 |
| 61 | Wright CJ, Dietze PM, Crockett B, Lim MS. Participatory development of MIDY (Mobile Intervention for Drinking in Young people). BMC Public Health [Internet] 2016;16:184. [doi: 10.1186/s12889-016-2876-5] |
| 62 | Zachary WW, Michlig G, Kaplan A, Nguyen NT, Quinn CC, Surkan PJ. Participatory Design of a Social Networking App to Support Type II Diabetes Self-Management in Low-Income Minority Communities. Proc Int Symp Hum Factors Erg Heal [Internet] 2017;6(1):37–43. PMID:31157286 |
