# Supplementary material for: Generative Participatory Design Methodology to Develop Electronic Health Interventions: Systematic Literature Review
Source: J Med Internet Res. 2020 Apr 27;22(4):e13780. doi: 10.2196/13780 (PMC7215492; doi:10.2196/13780)
Supplement: Multimedia Appendix 3 [file jmir_v22i4e13780_app3.docx]

**Multimedia Appendix 3: Reporting on stakeholders, tools and outcomes**

| Author | Health domain | eHealth type | Aim describe PD | Stakeholder recruitment | Stakeholder Management | Tools | eHealth evaluation outcome measures | PD evaluation outcome measures | Reporting score^a^ |
| --- | --- | --- | --- | --- | --- | --- | --- | --- | --- |
|  |  |  |  |  |  |  |  |  |  |
| Ahmed | Heart disease | mHealth | √ | √ | √ | √ | - | - | 34 |
| Aljaberi | Maternal care | mHealth | - | √ | √ | √ | - | - | 23 |
| Allin | Nervous system | Web-based tool | √ | - | - | √ | - | - | 12 |
| Alsem | Physical disability | Web-based tool | - | - | √ | √ | - | - | 13 |
| Beaudry | Child care | Chatbo | - | √ | - | √ | √ | - | 13 |
| Berg | Diabetes | Web-based tool | - | - | - | √ | - | - | 11 |
| Bjerkan | Mental health | Web-based tool | - | √ | √ | √ | - | - | 12 |
| Bravo | Cancer | Emails | - | - | - | √ | √ | - | 17 |
| Castensoe-Seidenfaden | Diabetes | mHealth | √ | √ | √ | √ | √ | - | 26 |
| Cheng | Mental health | mHealth | - | √ | - | √ | √ | - | 22 |
| Chomutare | Diabetes | mHealth | - | - | - | √ | - | - | 11 |
| Coyne | Multiple | Website | - | - | - | √ | √ | - | 15 |
| Crosby | Blood disease | Web-based tool | - | - | - | √ | √ | - | 15 |
| Danbjorg | Arthritis | mHealth | - | √ | √ | √ | - | - | 25 |
| Das | Nutrition | Web-based tool | √ | √ | √ | √ | √ | - | 33 |
| Davis^c^ | Asthma | Mobile phone app | √ | - | - | √ | √ | - | 20 |
| Easton | Lung disease | Chatbot | - | √ | - | √ | - | - | 17 |
| Gardsten | Diabetes | - | √ | √ | √ | √ | - | - | 31 |
| Garzo | Parkinson | Web-based tool | √ | - | - | √ | √ | - | 25 |
| Giroux | Elderly care | - | - | √ | √ | √ | - | - | 28 |
| Gonsalves | Mental Health | mHealth | - | √ | - | √ | - | - | 30 |
| Gordon | Mental Health | mHealth | - | √ | - | √ | - | - | 17 |
| Grenha Teixeira | Multiple | Electronic Health Record | - | - | - | √ | √ | - | 21 |
| Grim | Mental health | Web-based tool | √ | √ | - | √ | - | - | 11 |
| Hasvold | Surgery | mHealth | √ | - | - | √ | - | - | 18 |
| Hetrick | Mental health | Mobile phone app | - | √ | √ | √ | - | - | 21 |
| Hobson | Nervous system | Telehealth | - | - | √ | √ | - | - | 18 |
| How | Nervous system | Tele-health | √ | √ | √ | √ | √ | √ | 34 |
| Iribarren | Alzheimer | - | - | √ | - | √ | - | - | 16 |
| Jakobsen | Oesteoporosis | mHealth | - | √ | - | √ | - | - | 30 |
| Jessen | Multiple | mHealth | √ | √ | √ | √ | - | - | 30 |
| Joensson | Heart disease | mHealth | - | √ | - | √ | √ | - | 24 |
| Konnerup | Aphasia | - | - | √ | √ | √ | - | - | 19 |
| Kristiansen | Heart disease | Website | - | √ | - | √ | - | - | 32 |
| LaMonica | Mental Health | Web-based platform | - | √ | √ | √ | √ | - | 30 |
| Lucero | Fall prevention | Web-based platform | √ | √ | - | √ | - | - | 36 |
| March | Mental health | Web-based tool | √ | - | - | √ | √ | - | 28 |
| Nielsen | Kidney transplantation | mHealth | √ | - | - | √ | - | - | 32 |
| Noergaard | Heart disease | Web-based tool | - | - | √ | √ | - | - | 19 |
| O'Brien | Elderly care | Web-based tool | √ | - | √ | √ | - | - | 36 |
| Ospinna-Pinnilos 2018 | Mental health | Web-based tool | √ | √ | √ | √ | √ | - | 22 |
| Ospinna-Pinnilos 2019 | Mental health | Web-based tool | - | √ | - | √ | - | - | 22 |
| Peters^b^ | Asthma | Mobile phone app | - | √ | √ | √ | √ | - | 20 |
| Phillips | Smoking | Web-based tool | - | √ | - | √ | - | - | 17 |
| Rassmus-Gröhn | Heat disease | - | - | - | √ | √ | - | - | 20 |
| Revenas | Parkinson | - | √ | √ | √ | √ | - | √ | 36 |
| Rochat | Elderly care | Web-based tool | √ | - | - | √ | - | - | 15 |
| Ruland 2006 | Cancer | mHealth | - | √ | √ | √ | - | - | 15 |
| Ruland 2008 | Child care | mHealth | √ | √ | √ | √ | √ | - | 35 |
| Ruland 2009 | Cancer | Web-based tool | √ | - | - | √ | √ | - | 8 |
| Ryu | Multiple | Elec-tronic health record | - | - | - | √ | √ | - | 14 |
| Scandurra | Multiple | Elec-tronic health record | - | - | - | √ | √ | - | 8 |
| Slomian | Maternal care | Not defined | - | √ | - | √ | - | - | 12 |
| Skeels | Cancer | NA | - | √ | √ | √ | - | - | 25 |
| Stalberg | Child care | Application | √ | √ | √ | √ | √ | - | 20 |
| Swallow | Elderly care | mHealth | - | √ | √ | √ | √ | - | 29 |
| Switsers | Mental health | mHealth | - | √ | √ | √ | - | - | 29 |
| Tang | Multiple | Web-based tool | - | - | - | √ | - | - | 22 |
| Terp | Mental health | Mobile phone app | √ | √ | √ | √ | - | - | 34 |
| VanHeerwaarden^b^ | Mental health | Web-based tool | √ | √ | √ | √ | √ | √ | 30 |
| Verbiest | Obesity | mHealth | √ | - | - | √ | - | - | 30 |
| Waller | Diabetes | Text message | - | - | √ | √ | - | - | 13 |
| Warnestal | Cancer | Web-based tool | - | - | - | √ | - | - | 15 |
| Wherton | Elderly care | Telehealth | - | - | - | √ | - | - | 19 |
| Whitehouse | Mental health | Web-based tool | - | - | - | √ | - | - | 15 |
| Wiljer ^b^ | Mental health | Web-based tool | √ | - | - | √ | - | - | 8 |
| Woods^b^ 2017 | Heart disease | mHealth | - | - | - | √ | - | - | 19 |
| Woods^b^ 2018 | Heart disease | mHealth | √ | - | - | √ | - | - | 23 |
| Woods^b^ 2019 | Heart disease | mHealth | - | √ | - | √ | - | - | 18 |

^a^Sufficiency of reporting score (max 40)

^b^Of the 69 studies, there are 65 unique eHealth products or services under development, Peters covers the same eHealth as Davis , Wiljer covers the same eHealth as VanHeerwaarden and three Woods studies cover the same eHealth.
