# Supplementary material for: Generative Participatory Design Methodology to Develop Electronic Health Interventions: Systematic Literature Review
Source: J Med Internet Res. 2020 Apr 27;22(4):e13780. doi: 10.2196/13780 (PMC7215492; doi:10.2196/13780)
Supplement: Multimedia Appendix 4 [file jmir_v22i4e13780_app4.docx]

**Multimedia Appendix 4: Stakeholder recruitment and management**

| **Stakeholder recruitment** | **Stakeholder management** | **Study** |
| --- | --- | --- |
| **Patients**: recruited via previous focus group from a large not-for-profit cardiology clinic in the Midwest. The device clinic, part of ambulatory care, provided a list of adult patients with implantable CRT devices. Criteria: A research nurse screened these records for patients with HF with reduced ejection fraction. Patients were contacted by phone by the research nurse and invited to participate in design sessions later along with an informal caregiver of their choosing. All participants received $40. | (1) As an ice-breaker moderator introduced the participants to the main design activity of a dashboard via a car dashboard (2) PowerPoint to explain key data points with metaphors of a stoplight (3) blank cards were provided to stimulate intuitive representations, participants were encouraged to write directly on the cards and change them later, (4) all participants were stimulated to walk around and look at others, (5) at final consensus round moderators addressed only inconsistencies among all teams and invited others to evaluate | Ahmed |
| **Experience experts**: Snowball sampling (email and in-person recruitment), Criteria: (1) Caribbean immigrant women South Florida, (2) low income, minimum wage (3) given birth to at least one child in the United States in the age range of 18 to 30 years and in the last 5 years. All other age groups were excluded because of higher risk birth complications that were outside the scope of this study, (4) English (5) basic knowledge of using cell phones and the Internet | Reassurance by researcher no right or wrong, no testing of skills, demonstration of simple examples to show expectations, to avoid intimidation | Aljaberi |
| *Core team***: Researchers:** one with spinal cord injury involved with online resources for spinal cord community (Project manager), one researcher human-computer interaction researcher**, Software developer**: via consumer advocacy group, with spinal cord injury**, Interaction designer**: via consumer advocacy group, with spinal cord injury  *Informants***: People with spinal cord injury:** via advocacy organizations: recruitment designed for diversity: geography, lived experience, level of injury**, Physical activity experts:** via SCI action Canada**, Dietitian**: via hospital**, Researchers:** via SCI Action Canada, Toronto Rehabilitation Institute, Parkwood institute**, Clinician**: via Parkwood institute | (NR) | Allin |
| **Parents:** via previous study, parent organization, Facebook and Twitter, outpatient department, parents also recruited in their own network  **Clinical researcher, healthcare professional,**  **ICT specialist**: (NR) | When in doubt feedback was discussed with parent representative until consensus reached | Alsem |
| **Researchers, healthcare professionals, context experts, web programmer, designer:** (NR) | (NR) | Berg |
| **Content experts, caregivers:** (NR) | (NR) | Billis |
| **Participants with disabilities**: via municipalities of testing sites, Criteria: (1) legal entitlement care (2) access to internet on cell phone and computer  **Care professionals, researchers, developer:** (NR) | Flat communication structure to enable (informal) contact with project team and to share stories and information | Bjerkan |
| **Patients**: Nurse clinicians and physicians recruited patients within their clinic, $25 to attend the introductory workshop, $50 for completing the post-project survey | (NR) | Boudry |
| **Physicians**: Purposive and convenience sampling adopter physician and non adopter physician, Can $ 200 gift card, Criteria: logging on activity | (NR) | Bravo |
| **Patients, parents, care professional**: Purposive sampling strategy was used to ensure variation in age, gender, age at onset of T1DM, and location of diabetes care.  Criteria young people (and parents): 14-22 years old, T1DM ≥1 year, no psychiatric disorders, in pediatric care or adult care  Criteria health care providers: diabetes team, adult or a pediatric and adolescent diabetes clinic and with ≥1 year experience working with young people with diabetes  **Designer, software developer, dietician, psychologist, social worker, journalist**: (NR) | Workshop participants were grouped by whether they were young people, parents, or health care providers. | ﻿Castensoe-Seidenfaden |
| **Context expert, researchers, designers**: convenience sampling: via posters, distributing postcards, and advertising in student mailing lists, asking affiliated organizations to assist in recruitment for locations not in Sydney. Reimbursement of Aus $50, user experience interview participants Aus $30 | (NR) | Cheng |
| **Patients, content expert, parents**: (NR) | (NR) | Chomutare |
| *Co-design group***: Adolescent and young adult content experts from three diseases:**  via email to participants previous project, promotion voluntary organizations, social media, $20 gift card.  *Advisory group***: Physician, nurse, parent, young adult, clinical psychologist, lecturer, web developer, digital technology expert:** (NR)  *Second advisory group***: Parents and members of Cystic Fibrosis, Diabetes and Heart Children, Children in Hospital:** (NR) | (NR) | Coyne |
| **Patients:** Adolescent and young adults via pediatric medical center, adult medical via letter, phone call, clinic appointments, or SCD-related events  **Healthcare providers**: (NR) | (NR) | Crosby |
| **Patients**: purposive sampling: via supervising physiotherapist  Criteria: experiences with training and interest in using an exercise app, speak Danish | Introduction to the workshop and role in designing the future app | Danbjorg |
| **Care professionals:** Convenient sampling, Criteria: experience with obesity treatment  **Patients:** Criteria: complete a weight reduction program  **Software developers and researchers:** (NR) | (1) To prevent professional led power imbalance in relationship to patients separate workshops were organized; (2) smaller breakout groups were organized for free discussion; (3) active facilitation for challenging topics and self-disclosure, and scope guidance | Das |
| **People with asthma:** via social media, university websites, flyers in community pharmacies, sporting clubs, university notice boards, approaching school nurses and asthma educators, organizations for young people | (NR) | Davis |
| **Patients**: via lung foundation: Criteria: individuals with self-reported diagnosis of COPD or chronic lung disease, medically stable, and with a self-reported experience of emotional symptoms in relation to their LTC, diagnosed mental health condition was not an inclusion criterion for taking part; however, mental health conditions in patients with physical comorbidities are common and often undiagnosed.  **Care professionals**: snowball sampling: Criteria: experience with patient group as a general practitioner, mental health professional, specialist nurse, occupational therapist, physiotherapist, or pulmonary rehabilitation specialist. | (NR) | Easton |
| **Patients:** purposive sample via health care centre and diabetes hospital clinic, recently diagnosed adults (≤ 3 years) and adults with experience of diabetes (≥ 5 years) were invited because they may have different desires for self-management, these participants were invited after focus group to participate in workshop | Main facilitator role: introduce, lead, and summarize the sessions; the assistant facilitator supported the main facilitator, contributed to dialogues, made observations, and notes to ask questions and give feedback. Facilitators’ responsibilities were also to structure the overall process and to take an active role in helping participants to suggest creative ideas without being dominant in anyway. | Gardsten |
| (NR) | (NR) | Garzo |
| **Care professionals:** ﻿representative sampling strategy based on importance of including all potential users in designing the tool: caregivers, service providers from community settings, and professionals from the health and social services network.  **Advisory committee**: researchers, caregivers, community workers, health and social service professional | Methods used in large groups or small subgroups, mixed subgroups used to cross perspectives, whereas division by type of co-designer was used when we wished to highlight the perception of caregivers, for each subgroup a moderator (a member of the team for each subgroup) monitored the conduct of the activity and the role of each participant. | Giroux |
| **Context experts:** via classroom announcements and teacher nominations, to capture different perspectives, equal quotas for age and gender were used. No specific eligibility criteria were used to select adolescents on the basis of current/prior experiences of mental health problems, proficiency in English, Hindi, or Konkani, and to provide informed assent and parental consent. | (NR) | Gonsalves |
| **Patients:** key members of the staff seen as potential champions of the project identified, clinicians asked to identify patient members with a history of perinatal depression and potential interest in participating. Compensation of $25 to $50, participants were also provided with a simple dinner. | (NR) | Gordon |
| **Psychiatry patients:** convenience sample via phone and email to community based mental health services and non-profit user organizations, Criteria: experience from using psychiatric services | (NR) | Grim |
| **﻿**(NR) | (NR) | Hasvold |
| **Young people:** via clinicians or coordinator of youth advisory group, and chain-referral sampling: people referred to website with information and expression of interest form, clinical psychologist contacted young people with eligibility criteria, reimbursement $30 per hour  **Researcher, designers, digital design student, clinicians**: (NR) | Facilitation for shared vision: clinician workshop before and after the workshop with young people | Hetrick |
| **Patients:** via word-of-mouth through association charity and research advisory group  **Informal caregivers, specialist nurse**: (NR) | (1) Facilitation by clinician and user-centered design specialist and telehealth user experience designer, (2) icebreaker session used puzzles and games to introduce the group and to establish aims and rules of the workshop | Hobson |
| **Health care professionals:** Criteria: > 19 years, > 5 years clinical experience in rehabilitation TBI outpatients, specializes cognitive rehabilitation on brain-injured population  **Researchers:** (NR) | (1) Clarification of technology concepts for clinicians, (2) time to practice design (fieldkits), (3) and being able to expand on conceptual ideas clinicians generated,(4) technology designers needed understanding of cognitive rehabilitation for deeper discussion, (5) fieldkit mediated dual-voice of clinicians of own experience and on behalf of clients | How |
| **Patients**: purposive sampling, enrolled in the caregiver registry who agreed to participate in future studies and were computer/Internet users. Criteria: adults (between age of 18 and 90), self-identifying Hispanic, caregiver of a family member or significant other diagnosed with ADRD who reported at least one memory/cognition and one daily functioning symptom. ﻿To facilitate future contact, the caregiver had to live in vicinity for 12 months following enrollment and have one relative or close friend also living in vicinity, physically able to provide care and no diagnosis of depression with psychotic features, suicidal ideation or attempt in the past 5 years. Zip codes were used to obtain a list of potential participant closest to the research location to reduce participant burden. ﻿Participants received $35 in compensation for time and transportation. | (NR) | Iribarren |
| The aim was to establish a team representing patients and stakeholders from all levels within the field of early detection of osteoporosis in women.  **Women and care professionals**: via Department of Endocrinology at a Danish University Hospital and the general practitioners (GPs) from nearby medical practices. | (NR) | Jakobsen |
| **Patients:** via 2 hospital educational centers in the northern and southern parts of Norway, as well as the youth council at a hospital in the Oslo region. Criteria: fluent in Norwegian, having a long-term health challenge, and being over the age of 16 years. | Workshops were made shorter to make participating less of a burden to chronic ill patients. In addition, shorter sessions would allow the participants to reflect on the content and concepts between the 2 gatherings. | Jessen |
| **Patients and care professionals**: via cardiology department at the hospitals in Viborg and Skive, and from the healthcare centers in Skive, Viborg, and Randers. | (NR) | Joensson |
| Focus on diverse age, gender, severity of aphasia (i.e., mild, medium, and severe), different degrees of mobility, and varied post-onset. As more men were interested in the project, motivation, engagement, and familiarity with technological tools were the criteria that was considered most important.  **Patients:** Criteria**:** diagnosed with aphasia, patient, participated in web-based rehabilitation before, computer at home with a newer graphic card and good bandwidth, been interested and willing to try new and untested rehabilitation methods. | Feeling comfortable in the team is fundamental for the participants to express  themselves, start with coffee/tea and introductions. For communication: the necessary time is given, talk about one subject at a time, communication partners are and appear present, everybody is treated on equal footing.  Video from a rehabilitation project for veterans with PTSD, was shown. It gave an impression of what an IVE and an avatar might be. | Konnerup |
| **Patients:** purposive sampling via specialist nurses, Criteria: > 18 years, understand Danish, treatment at heart failure clinic  **Designer, Researcher:** (NR) | (NR) | Kristiansen |
| **Content experts, informal caregivers**: via postcards, A3/A4 posters, social media, to avoid perceived coercion, recruitment was passive as potential participant needed to contact the Research Project Manager.  Criteria: aged 16 years or older and eligible for Open Arms services (all current and former ADF personnel who have at least 1 day continuous full-time service are eligible for care through Open Arms; having met this criterion, their family members are also eligible for care)  **Care professionals and government representatives**: via relevant Open Arms Centre managers in each of the 5 regions. | Detailed information provided about the study before attending a participatory design workshop and upon arrival at the workshop. At the beginning of each workshop, the facilitators provided the participants with an opportunity to ask questions and clarify details of the research before providing written informed consent. All workshops were coordinated by at least 2 facilitators, 1 of whom was a mental health professional whose role was to respond to any participant concerns or distress as a result of the subject matter. | LaMonica |
| **Context experts:** community-dwelling older adults, older adults self-reported whether they had a history of falling, not eligible for the study if they were not able to walk independently with or without an assistive device, had attended computer/Internet training and reported weekly internet access. | (NR) | Lucero |
| **Children:** (NR) | (NR) | March |
| (NR) | (NR) | Nielsen |
| **Patients:** aim diverse age, sex, duration with heart disease, and communicative skills, sufficient motivation, capabilities to engage actively,  **Physicians and nurses**: to ensure ownership (recruitment stopped during study due to lack of time of physicians)  **System architects, researchers, observers:** (NR) | (1) prior to each workshop detailed script for moderators, observers and system architects (2) main responsibility of moderators to facilitate hands-on exercises and discussions, system architects role to present concept and assist with technical issues, one moderator facilitated overall procedure | Noergaard |
| **Older adults:** purposive sample, local older people’s forum, aim for different stages of retirement transition and socioeconomic background  **Health and social care professionals**: voluntary sector and public health organizations related to wellbeing of people in retirement  **Researchers**: from range of disciplines improving wellbeing older people: design, behavior change, public health, physical activity, nutrition and dietetics and social gerontology | (1) small groups (2) facilitators guided structure and time (3) support of a creativity facilitator for hand-drawn prototypes | O’Brien |
| **Young people:** flyers, informing health professionals, reception staff, headspace groups. Criteria: age 16-25, access to internet via mobile phone, tablet, desktop, laptop, gift voucher  **Health professionals**: senior management called for participation | Mental health professional was present during workshop in case of experienced psychological distress | Ospinna-Pinnilos |
| ﻿ **Context experts, informal caregivers, care professionals**: recruitment via headspace groups; poster and postcard; Facebook; use of organizational social media channels; universities, institutes of technical and further education, language schools, and vocational and training ﻿institutes; and cooperation with Spanish-speaking consulates in Australia.  Gift vouchers were given. | (NR) | Ospinna-Pinnilos |
| **Young people:** convenience sampling, via social media, university websites, community pharmacies, sporting clubs, high school nurses, asthma educators, organizations for young people, gift voucher $50. Criteria: age 15-24, residency Australia, doctor diagnosed asthma | User-experience specialist guided participants | Peters |
| **Content experts:** via gatekeepers at AIDS Service Organizations in an effort to bring together average GMLWH who smoke from among members of the organization. ﻿Criteria: self-describing gay and HIV positive, expressing desire to quit smoking or having quit within the prior 6 months, being older than 19 years of age, and being available for the participatory design session. Can$50 honorarium is provided. | (NR) | Phillips |
| (NR) | Participants were introduced to the ideas behind the project and a presentation was given by an expert user who uses technology. | Rassmus-Gröhn |
| **﻿﻿Patients:** via patient association, Criteria: ability to communicate in Swedish, availability for participation in all 4 workshops. Variation in age, gender, and years since diagnosis were also considered  **Care professionals**: via neurologist member of the research group sent emails | Each workshop was introduced by one of the researchers who informed participants about the aim and structure of the day and summarized previous achievements. | Revenas |
| **People 50-80 years old:** (NR) | (NR) | Rochat |
| **﻿Content experts**: via elementary school via letters, gift NOK 500 after the last session for participating**.** | 4 adults trained as participant or observer participated in each session, helped to form design ideas according to the purpose of the system and its ramifications. Adults were responsible for showing interface and navigation examples and helping the children understand what is technically feasible. | Ruland 2006 |
| **Content experts**: via elementary school via letters, gift NOK 500 after the last session for participating**.** Criteria: is comfortable with and is usually active in group activities, uses computers as an educational tool and to play games, is creative, e.g. likes to draw or enjoys building things, if there was a particular reason for why the child wanted to participate. | 4 adults trained as participant or observer participated in each session, helped to form design ideas according to the purpose of the system and its ramifications. Adults were responsible for showing interface and navigation examples and helping the children understand what is technically feasible. | Ruland 2007 |
| **Health care professionals:** (NR) | (NR) | Ruland 2009 |
| **Clinicians, Informatics specialists, developers, user experience specialists:** (NR) | (NR) | Ryu |
| **General practitioners, nurses, home help service personnel:** (NR) | (NR) | Scandurra |
| **Parents:** via social networks and website and online platform, and already in contact with research team. Criteria: parent of child < 2 years, no multiple gestation pregnancy, no fetal death in utero, not very premature childbirth (<34 weeks of gestation), and no fetal pathologies.  **Midwives, gynecologist, psychologist, medical-social worker, technology experts:** Criteria**:** involved in postnatal period from Belgian office of Birth and Childhood, no exclusion criteria | (NR) | Slomian |
| **﻿Patients:** via flyers, emails, and online postings, compensations of $110 for attending. | ﻿Not to overly burden participants we asked for a shorter commitment from a larger number of participants. ﻿The group started with a blank slate. We chose not to begin by introducing them to the existing technology because we did not want to influence participants’ priorities or constrain their ideas. | Skeels |
| **Children:** via preschool (PS), primary health care clinic (PHCC) and paediatric outpatient unit at a hospital (POU). Criteria: speaking Swedish | Age appropriateness assessed according to motor skills and knowledge skills | Stalberg |
| **Content experts**: recruited if willing to discuss mobility problems and explore how technology, such as mobile phones and tablet devices, may be used to support people to get out and about in their local area, no previous experience with mobile phones or tablet devices was necessary. £25 gift voucher provided | Workshops began with a presentation by the research team on the outcomes of a previous study, which adopted a photo diary method to explore the experiences of older adults and elicit requirements for new technologies. The presentation helped to introduce participants to the overall objectives and the purpose of the participatory design workshops. | Swallow |
| **People with bipolar disorder:** via Flemish patient network and self-help organization> Criteria: received diagnosis from health professional, received treatment, > 18 years, positive screening according to Mood Disorder Questionnaire | (1) Focus groups conducted by trained clinicians under supervision of the lead researchers, (2) small groups due to strong emotional impact | Switsers |
| **Project lead, project manager, developers, business analyst, physician lead, nursing lead, learner:** (NR) | (NR) | Tang |
| **Young adults:** purposive sampling strategy via outpatient clinic, age 18-34, suffer from severe symptomatology. Criteria: willingness to share information, ability to provide written consent, ability to talk about problems or understand Danish  **Health care practitioners:** via outpatient clinic | Facilitation supported by graphics | Terp |
| (NR) | (NR) | Timmerman |
| **﻿Context experts:** via preexisting Thought Spot social media accounts, flyers, academic departments and student organizations, existing connections within the Thought Spot student advisory group and the research team. Participants received a small honorarium and public transit tokens for attending each workshop, and food was served at all workshops. | ﻿Dividing participants into smaller groups during discussions helped ensure that most participants were able to contribute. ﻿A world café exercise was used during the final Thought Spot workshop, which focused on confirming our findings from the previous four workshops and eliciting diverse perspectives. World cafés involve small groups cycling through a series of questions at different stations and building on the answers of the previous groups. This method attempts to obtain diverse perspectives, rather than to achieve consensus, to better understand the overall experience. | VanHeerwaarden |
| **Computing student, Pediatrician:** (NR) | (1) Distinction between conceptual and physical design was made to help clinician express ideas regardless of whether or not implementation is possible or not (2) close collaboration | Waller |
| **Children, researchers:** (NR) | (NR) | Warmestal |
| **End-users, service providers, technology industry representatives:** (NR) | (NR) | Wherton |
| **Youth:** design student, youth via advisory committee  **Physician, nurses, social workers, developmental psychologist:** (NR) | (NR) | Whitehouse |
| **Youth:** (NR) | (NR) | Wiljer |
| **Patients, healthcare professionals, informal caregivers, app developer, researcher:** (NR) | (NR) | Woods 2017 |
| (NR) | (NR) | Woods 2018 (Partnering in digitial Health) |
| **Health care professionals:** (NR) | (NR) | Woods 2018 (Conceptual design) |
| **﻿Care professionals, patients, informal caregivers:** via a large metropolitan tertiary hospital campus specializing in cardiac care | (NR) | Woods 2019 |
